# Cuproptosis Inducers in Cancer Therapy: State of the Art and Challenges

**DOI:** 10.1002/tcr.70179

**Published:** 2026-06-22

**Authors:** Chiara Ragusa, Valentina Oliveri

**Affiliations:** ^1^ Dipartimento di Scienze Chimiche Università degli Studi di Catania Catania Italy

**Keywords:** cancer, cancer therapy, cancer resistance, cause of death, copper‐based nanomaterials, copper ionophores, cuproptosis, programmed cell death

## Abstract

Cancer continues to be the primary cause of death despite significant progress in medicine, and finding effective therapies remains a challenge. New treatments are needed to minimize the harmful effects on the body and increase the selectivity of drugs. These therapies must also overcome cancer cell resistance and prevent metastasis. Metal‐based drugs are becoming increasingly crucial for treating tumors, and copper ion‐based systems and nanoparticles have been identified as having unique properties and anticancer potential. Given the key role exerted by Cu in the etiology, severity, and progression of cancer diseases, it could be a vulnerable point to target for hindering cancer development. After thoroughly analyzing what is known about the mechanism of cancer cell death through a Cu‐dependent mechanism, known as cuproptosis, and its potential links with ferroptosis, we report some systems that, when coadministered with copper ions, can trigger this type of cell death. This study investigates the effectiveness of cuproptosis inducers against various types of cancers. Are these cuproptosis inducers effective in combating cancer? What limitations or disadvantages might be associated with their use? This article aims to answer these questions based on the current knowledge in this evolving area of cancer research.

## Graphical abstract

Cuproptosis is emerging as a distinct copper‐dependent cell death pathway, highlighting copper as a potential metabolic vulnerability in cancer. This review examines how coordination chemistry, redox regulation, and nanomaterial design shape Cu reactivity and therapeutic outcomes. Advances in ionophores and nanoplatforms are discussed alongside key challenges for translating cuproptosis into precision oncology.

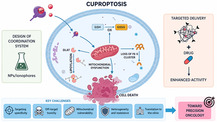

## 1 Introduction

Copper (Cu) is one of the essential metals in the human body. It is usually present in the system as a Cu‐binding protein, while only a minimal amount of Cu is in the free form [1]. In biological environments, Cu occurs in two main oxidation states, Cu(I) and Cu(II). The low redox potential of the Cu(II)/Cu(I) couple underlies its remarkable redox versatility and ability to participate in electron‐transfer processes. Throughout this review, Cu denotes Cu ions in general, except when reference to a specific oxidation state is required. Despite the limited amount of Cu, it plays a crucial role in various physiological processes, including mitochondrial respiration, synthesis of biomolecules, and iron absorption in the human organism [2].

**FIGURE 1:**
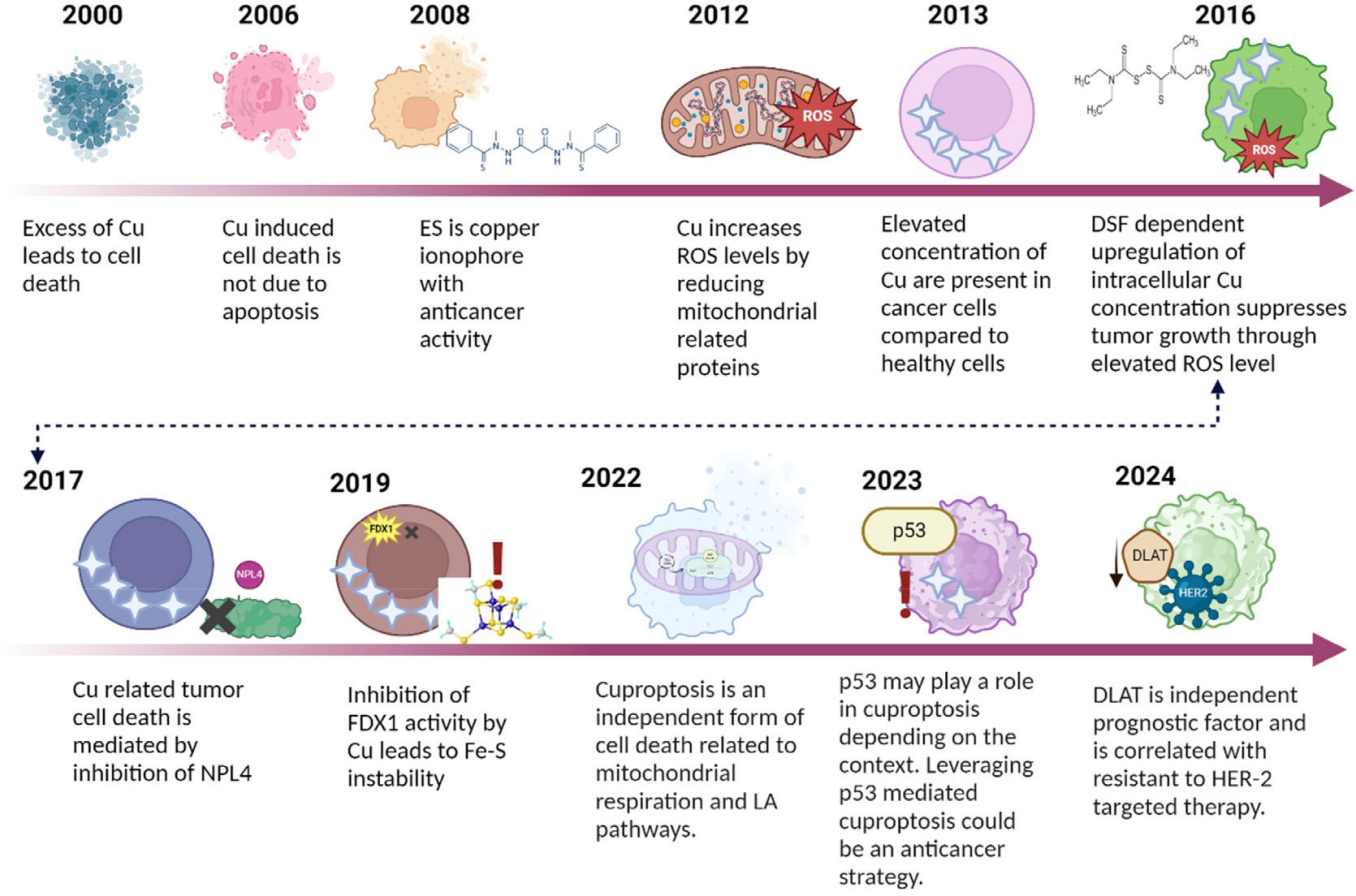
Timeline of the last 25 years highlighting discoveries related to Cu and cancer. This timeline depicts the historical events that contributed to the discovery of cuproptosis and the advances in oncological research related to Cu‐associated cell death.

It also plays a role as a dynamic signaling molecule and allosteric regulator that affects vital cellular pathways. The maintenance of Cu homeostasis is crucial for the normal functioning of cells. An excess of this metal can lead to metabolic abnormalities and toxicity as it can directly damage cell components and disrupt the cell redox balance, causing DNA damage and ultimately leading to cell death [3, 4]. Scientists have long been fascinated by how Cu induces cell death (Figure 1), and Tsvetkov et al. were the first to describe and name this distinct process as cuproptosis [5]. This phenomenon involves Cu accumulation in mitochondria, leading to DLAT aggregation and proteotoxic stress, alongside the loss of iron–sulfur cluster (ISC, Fe–S) proteins [6, 7].

**FIGURE 2:**
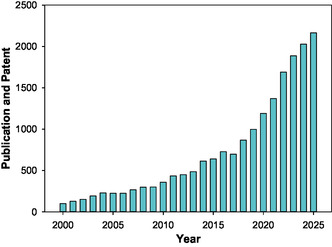
Graphical timeline of Cu and cancer research over the past 25 years. Data collected on February 23, 2026.

Before Tsvetkov's article, there was evidence suggesting a connection between Cu and cancer. Researchers investigated the role of Cu in cancer pathogenesis and its potential as a target in anticancer therapy. Figure 1 outlines all the key steps that we believe marked the history of the discovery of the link between Cu and cancer. However, since the mechanism of action was shrouded in mystery, enthusiasm was somewhat limited until a few years ago, when cuproptosis began to be discussed; from that moment on, the number of publications on the subject has increased almost exponentially (Figure 2). Today, it is believed that Cu‐based therapy could be a promising approach for effectively inhibiting tumors that are resistant to chemotherapy.

Cuproptosis has become a key mechanism across various cancer types, and increasing evidence connects the expression of important cuproptosis‐related genes to tumor prognosis. Understanding these links is crucial not only for grasping tumor biology but also for guiding the development of next‐generation therapeutic strategies [8].

In this study, we examine the mechanism of cuproptosis, highlighting the molecular features that distinguish it from other regulated cell death pathways, including ferroptosis. Although much of the current literature has centered on biological validation and therapeutic implications, our perspective places particular emphasis on the chemical foundations of cuproptosis induction. We focus on the rational construction of Cu ionophores and Cu‐based platforms, discussing how metal oxidation state, coordination environment, and ligand architecture govern intracellular Cu trafficking and ultimately determine biological outcomes. By integrating mechanistic understanding with a construction‐oriented approach, this review aims to provide a chemically grounded guide for the development of next‐generation Cu‐based systems targeting cuproptosis in cancer therapy.

### 1.1 Crosstalk Between Copper and Cancer

Interestingly, numerous studies have reported altered Cu levels in the bloodstream and tumors across different cancer types. In particular, elevated Cu concentrations have been observed in the serum and tissues of patients with malignancies such as gallbladder, colorectal, and thyroid cancers [3, 8, 9, 10, 11].

In the past few years, Cu‐dependent cancer cell proliferation and death have given rise to the new concept of cuproplasia and cuproptosis. Tumor cells exhibit a phenomenon known as cuproplasia, defined as the pathological dependence of tumors on Cu ions that support malignant transformation, proliferation, and metastasis [9]. Tumor cells rely more heavily on Cu for their metabolism than normal cells. Indeed, Cu plays a central role in cancer metabolism and progression by acting as a cofactor in multiple cellular pathways. In mitochondria, this metal is required for the activity of cytochrome c oxidase, an enzyme responsible for the final step of oxidative phosphorylation and ATP production [12]. Consequently, malignant cells show a greater reliance on Cu compared with nonproliferating cells. Furthermore, Cu trafficking proteins, such as antioxidant protein 1 (ATOX1), Cu‐transporting ATPase 1 (ATP7A), and lysyl oxidase (LOX), facilitate extracellular matrix remodeling and metastatic spread [11, 13]. Cu also regulates autophagy through the activation of UNC‐51–like kinases 1 and 2 (ULK1 and ULK2), thereby providing adaptive advantages under metabolic and proteotoxic stress [14]. In addition, Cu promotes angiogenesis by directly activating proangiogenic mediators, including vascular endothelial growth factor (VEGF), fibroblast growth factor 2 (FGF2), and interleukin‐1 (IL‐1) [7]. Nevertheless, this dependence on Cu also represents a therapeutic vulnerability. Excessive disruption of Cu homeostasis can trigger cuproptosis. This insight has motivated the development of Cu ionophores and Cu‐based nanomaterials designed to selectively elevate intracellular Cu levels in tumors, effectively shifting the role of Cu from supporting cuproplasia‐driven proliferation to inducing cuproptosis‐mediated tumor suppression.

### 1.2 Cuproptosis: A Type of Cell Death

**FIGURE 3:**
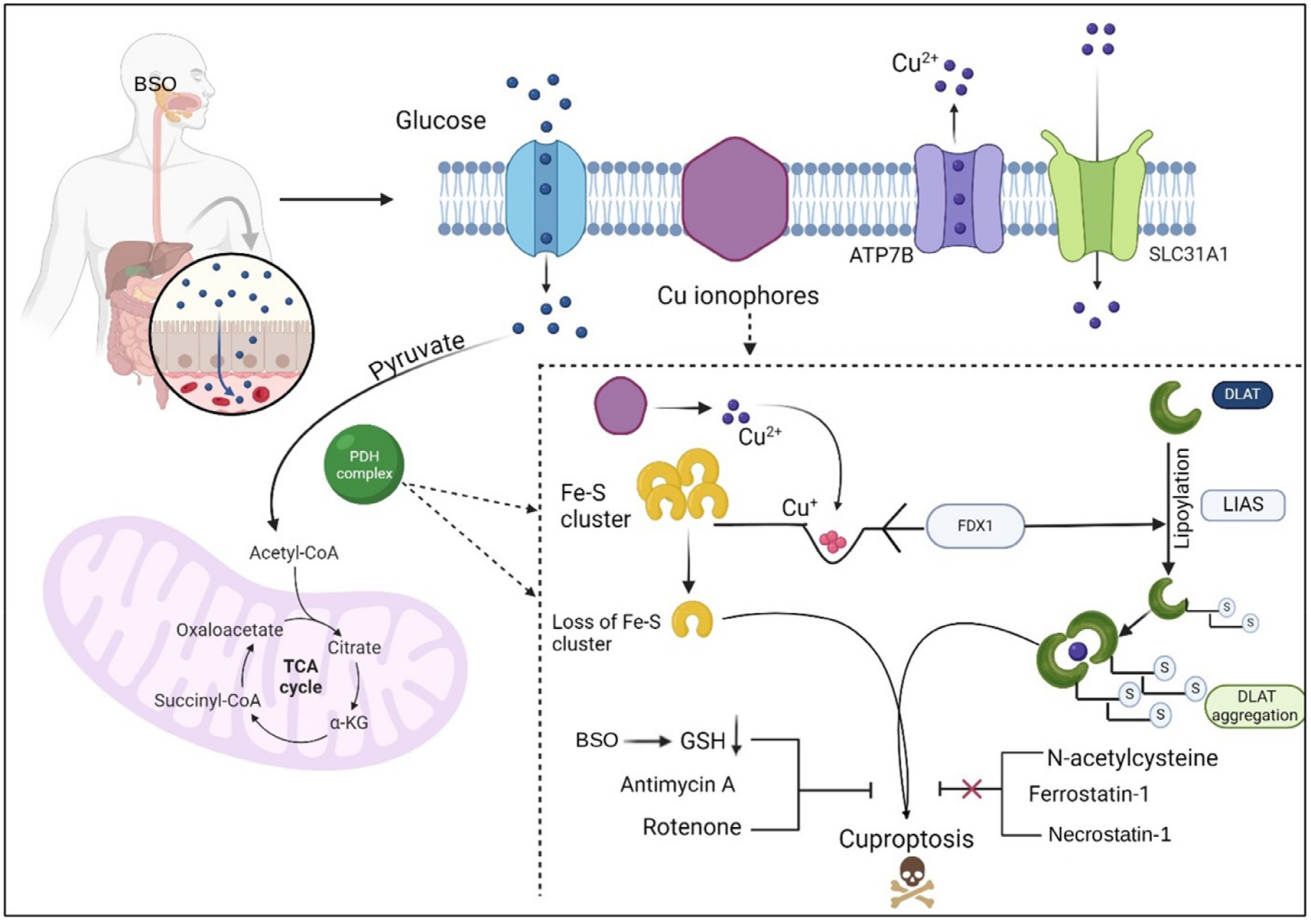
Schematic representation of the mechanism of cuproptosis. Glutathione (GSH) can prevent cell death caused by excess Cu. Conversely, buthionine sulfoximine (BSO) facilitates cuproptosis by reducing GSH levels. Additionally, the mitochondrial pyruvate carrier (MPC) inhibitor UK5099, along with inhibitors of ETC complexes I and III, such as rotenone and antimycin A, can mitigate the effects of cuproptosis. Inhibitors (ferrostatin‐1, necrostatin‐1, N‐acetyl cysteine) targeting apoptosis, necrosis, ferroptosis, and oxidative stress pathways are ineffective against Cu‐induced cell death.

Cuproptosis is a form of cell death distinct from other known mechanisms, such as apoptosis, necrosis, pyroptosis, and ferroptosis. Tsvetkov and colleagues identified a Cu‐dependent mode of cell death while investigating the anticancer activity of elesclomol (ES), a Cu ionophore [13]. Well‐known inhibitors of apoptosis (Z‐VAD‐FMK or BAK/BAX knockout), ferroptosis (ferrostatin‐1 or liproxstatin‐1), or necroptosis (necrostatin‐1) fail to prevent the cell death triggered by Cu ionophores (ES), indicating that these canonical mechanisms are not involved [5]. Remarkably, glutathione (GSH) reduces the toxicity of Cu ionophores, whereas other antioxidants (N‐acetylcysteine, α‐tocopherol, and ebselen) do not confer protection, indicating that cuproptosis is not primarily driven by ROS generation. Additionally, using Cu chelators like tetrathiomolybdate (TTM) can prevent or reverse the activation of cuproptosis, underscoring the specificity of Cu in this process. Furthermore, NCIH2030 lung cancer cells grown in galactose (which forces reliance on mitochondrial respiration) were more sensitive to the effects of Cu–ES than cells cultured in glucose (which favors glycolysis) [5]. Rotenone and antimycin A, inhibitors of respiratory chain complexes I and III, respectively, UK5099, an inhibitor of mitochondrial pyruvate uptake, markedly reduced cuproptosis. Real‐time oxygen consumption assays revealed that basal and ATP‐linked respiration were not affected by Cu–ES treatment and hypoxic conditions (1% O_2_), which shifted cells toward glycolysis, reducing the effects of the treatment [5]. These findings indicate that cuproptosis is tightly related to mitochondrial respiration and is mechanistically distinct from ferroptosis (coupled to glucose uptake and pyruvate oxidation). Unlike them, cuproptosis is closely linked to the cellular processes involved in mitochondrial metabolism. As stated in the introduction, it requires Cu accumulation and proteotoxic stress, leading to cell demise (Figure 3). Cuproptosis is also accompanied by a significant generation of the radical OH as demonstrated through an ultrasensitive and selective •OH fluorescence probe based on a coumarin–dihydroquinoline scaffold [15]. Cells take up Cu ions through Cu transporters like CTR1. Under normal conditions, the levels of Cu within cells are tightly regulated by proteins such as ATP7A and ATP7B, which facilitate Cu export and storage [16]. However, in cuproptosis, there is an abnormal accumulation of Cu within the cell, especially within the mitochondria. This can occur due to an increase in Cu import, a decrease in export, or a disruption in intracellular Cu storage. Ferredoxin 1 (FDX1) [17] is a key player in cuproptosis. It is a small protein containing a 2Fe–2S center and serves two primary functions in cuproptosis: first, it regulates protein lipoylation, though the exact mechanism behind this is still not fully understood; second, it facilitates the reduction of Cu(II) to Cu(I), making it more reactive and prone to binding with specific proteins [18, 19]. This enhanced Cu activity is critical for initiating the binding of Cu to lipoylated proteins, which are covalently modified with lipoic acid and essential for mitochondrial oxidative metabolism. Experiments have also shown that the role of Cu in binding to lipoylated proteins and causing mitochondrial dysfunction is unique, as other metals like iron or zinc do not induce the same effects.

Cu ionophores, small molecules that facilitate the transport of Cu ions across cellular membranes, can bypass the usual regulatory mechanisms and lead to a rapid and uncontrolled increase in intracellular Cu levels. As a result, they exacerbate the accumulation of Cu within the mitochondria. Once inside the mitochondria, the excess reactive Cu, due to Cu ionophores and influenced by FDX1, binds to specific lipoylated proteins, including dihydrolipoamide dehydrogenase (DLD), dihydrolipoamide succinyl transferase (DLST), and dihydrolipoamide acetyltransferase (DLAT) [20, 21]. Lipoylation is a post‐translational lysine modification where lipoic acid attaches to enzyme complexes, such as those in the tricarboxylic acid (TCA) cycle. These enzymes depend on lipoylation for their activity. DLAT and DLST, as essential subunits of the α‐ketoglutarate dehydrogenase complex, are crucial regulators of the TCA cycle [22].

Cu binding to lipoylated proteins induces their aggregation, leading to TCA cycle impairment, loss of Fe–S cluster proteins, and proteotoxic stress. Rather than causing cell death through loss of protein function, Cu(I) promotes oligomerization of these proteins, triggering proteotoxic stress and subsequent cell death [23]. This process disrupts mitochondrial respiration and energy production, contributing to reactive oxygen species (ROS) generation and mitochondrial dysfunction, including loss of membrane potential. Unlike apoptosis (caspase‐dependent) or ferroptosis (lipid peroxidation‐driven), cuproptosis is primarily driven by Cu‐induced mitochondrial damage, with FDX1 and Cu ionophores playing central roles [24]. These events collectively culminate in cell death.

However, many unanswered questions regarding cuproptosis remain to be studied further. One of the key gaps in our understanding of cuproptosis is the need for a comprehensive description of its characteristic manifestations. We have yet to determine whether cuproptosis is accompanied by distinct or sequential morphological changes, and the specific molecular or cellular alterations that occur postinduction of cuproptosis remain unidentified. This limited understanding complicates the identification and reliable assessment of cuproptosis in biological systems.

### 1.3 Cuproptosis Versus Ferroptosis

Ferroptosis is a regulated form of cell death driven by iron‐dependent oxidative stress and the consequent accumulation of lipid peroxides [25]. It is characterized by the depletion of GSH and the inactivation of GSH peroxidase 4 (GPX4), which leads to uncontrolled lipid peroxidation and membrane damage [26]. Cells undergoing ferroptosis display unique morphological features, such as condensed mitochondria with reduced cristae, but they do not show the nuclear changes typical of apoptosis (e.g., chromatin condensation) [27].

**FIGURE 4:**
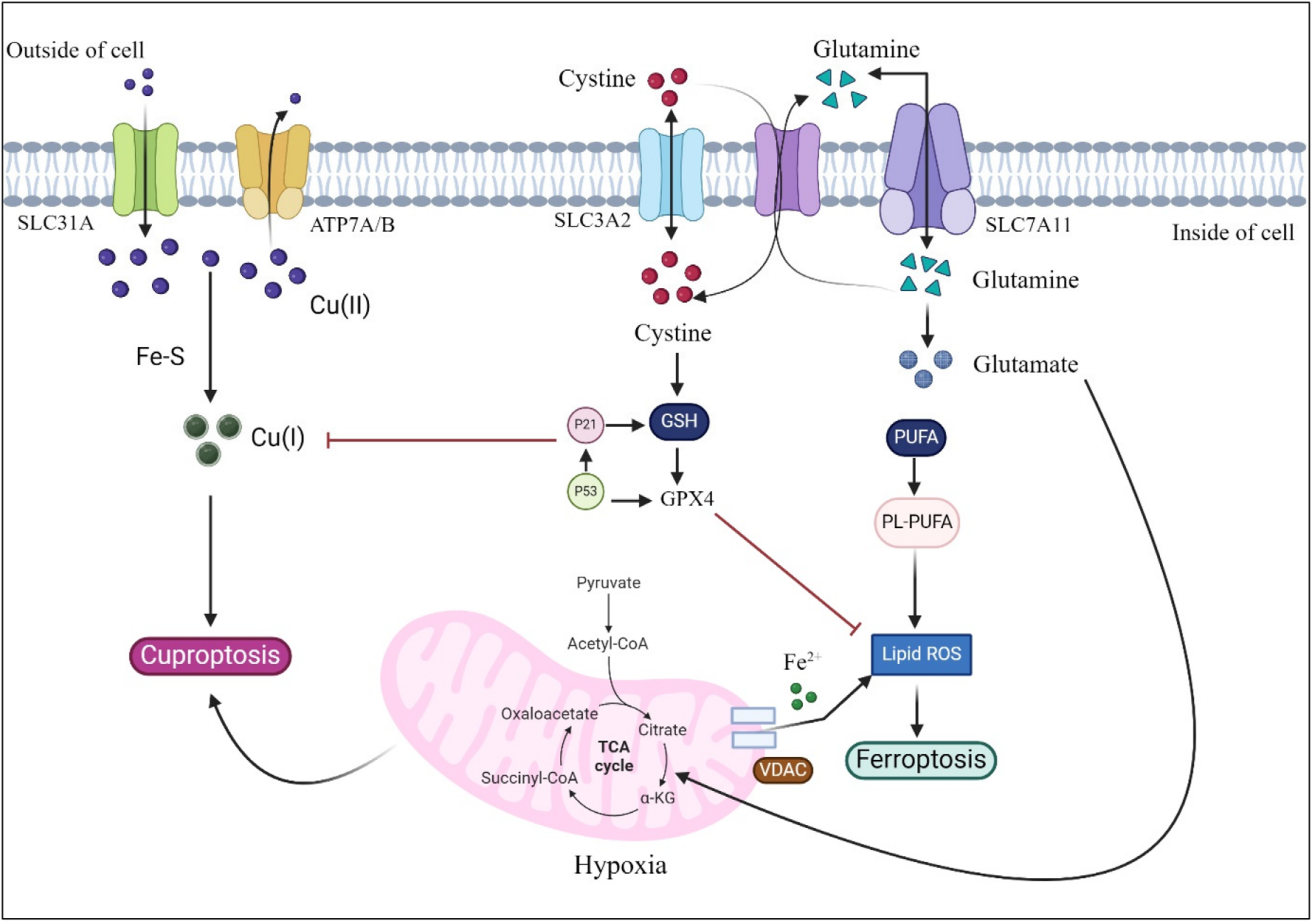
Relationship between ferroptosis and cuproptosis: (i) GSH can suppress ferroptosis by neutralizing ROS; elevated Cu levels activate the tumor suppressor gene p53, which can promote cuproptosis by enhancing mitochondrial activity and simultaneously induce ferroptosis by downregulating SLC7A11 (a membrane transporter of cystine), thereby disrupting cystine uptake, a precursor of GSH, and impairing antioxidant defenses (right side). (ii) Cu buildup triggers cuproptosis through FDX1‐mediated mitochondrial proteotoxic stress within the TCA cycle (left side). (iii) Although mechanistically distinct, both ferroptosis and cuproptosis, being mitochondria‐associated, are highly sensitive to hypoxic conditions (middle part). Colored channels in the schematic represent protein transporters, and colored dots represent the key ions. Black arrows show activation pathways, while red lines denote inhibitory mechanisms.

Cuproptosis is mainly triggered by intracellular Cu accumulation and is closely linked to mitochondrial metabolism. It occurs when Cu binds to lipoylated components of the TCA cycle. Unlike ferroptosis, lipid peroxidation is not the primary driver of cuproptosis (Figure 4).

Despite these differences, ferroptosis and cuproptosis share significant similarities (Table 1). Both processes are tightly connected to mitochondrial function and cellular redox homeostasis [28]. In particular, GSH represents a critical convergence point: its depletion promotes ferroptosis by inhibiting GPX4 activity, while in cuproptosis, it contributes to Cu toxicity by reducing cellular antioxidant defenses and increasing the accumulation of Cu‐bound proteins. Additionally, both pathways are associated with high levels of ROS, although ROS act as the primary driving force in ferroptosis and as secondary contributors in cuproptosis.

**TABLE 1:** Comparative analysis of cuproptosis and ferroptosis: molecular mechanisms, regulators, and distinguishing features.

Feature	Cuproptosis	Ferroptosis
Metal dependency	Cu‐dependent	Iron‐dependent
Primary trigger	Intracellular Cu accumulation and binding to lipoylated proteins	Lipid peroxidation driven by iron‐catalyzed ROS
Key molecular targets	Lipoylated components of the TCA cycle (e.g., DLAT)	Polyunsaturated fatty acids in membrane phospholipids
Core mechanism	Cu binds to lipoylated mitochondrial proteins → protein aggregation → loss of Fe–S cluster proteins → proteotoxic stress	Iron promotes Fenton reaction → lipid peroxides accumulate → membrane damage
Cellular localization	Primarily mitochondrial	Primarily cellular membranes
ROS involvement	Indirect; secondary consequence linked to mitochondrial dysfunction and proteotoxic stress	Direct; central role of lipid ROS accumulation
Key regulators	FDX1, lipoic acid pathway enzymes	GPX4, SLC7A11 (system Xc^‐^), ACSL4
Morphological features	Protein aggregation visible, mitochondrial dysfunction	Condensed mitochondria, reduced cristae, membrane damage
Inhibition strategies	Cu chelators (e.g., tetrathiomolybdate)	Lipophilic antioxidants (e.g., ferrostatin‐1), iron chelators
Shared features	Metal‐dependent regulation; mitochondrial involvement; ROS‐associated stress	Metal‐dependent regulation; mitochondrial involvement; ROS‐associated stress
Overall distinction	Protein aggregation–driven cell death	Lipid peroxidation–driven cell death

In vitro and in vivo experiments have shown that cotargeting ferroptosis and cuproptosis can significantly enhance anti‐cancer effects, offering a promising approach to overcoming resistance to conventional therapies [29]. Furthermore, some Cu‐based systems have shown the ability to activate ferroptosis or amplify its effect [30, 31]. Below are some examples:

(i) Treatment with the disulfiram–Cu (DSF–Cu) complex induces the activation of ferroptosis in triple‐negative breast cancer (TNBC). In particular, DSF–Cu treatment causes marked mitochondrial atrophy and is accompanied by an increase in intracellular iron, lipid ROS, and malondialdehyde, alongside a decrease in GSH levels. Collectively, these features suggest ferroptosis as the underlying mechanism of cell death. Moreover, transcriptome analysis revealed the activation of the ferroptosis signaling pathway, with the upregulation of heme oxygenase‐1 (HMOX1), a regulator of redox homeostasis, confirming the prominent role of ferroptosis in DSF–Cu‐mediated cytotoxicity [32]. In addition to its effects on TNBC, DSF–Cu was found to induce a ferroptotic mechanism in nasopharyngeal carcinoma (NPC) [33], urinary bladder cancer (UBC) [34], multiple myeloma (MM) [35], to name a few.
(ii) A water‐soluble Cu(II) complex, [Cu(DPQ)(Gluc)]·2H_2_O, obtained with D‐gluconic acid as an auxiliary ligand and DPQ (pyrazino[2,3‐f[1,10]phenanthroline) as an aromatic ligand displayed anticancer activity superior to that of cisplatin, against hepatocellular carcinoma (HCC, HepG2) cells. In vivo models, the complex significantly inhibited tumor growth. The ability of this complex has been attributed to the synergistic contribution of apoptosis (ROS generation, mitochondrial dysfunction, cell cycle arrest, caspase activation) and ferroptosis (lipid peroxide accumulation, GPX4 inhibition) [36].
(iii) Self‐assembled Cu‐alanine nanoparticles (CACG) coloaded with glucose oxidase (GOx) and cinnamaldehyde (Cin), have shown promising results in inducing ferroptosis and stimulating antitumor immunity [37]. In vivo, CACG effectively inhibited 4T1 tumor growth without systemic toxicity. As for the mechanism of action, CACG delivers Cu(II), Cin, and GOx into tumors. Cin depletes GSH via Michael addition, while Cu(II) is reduced to Cu(I) further reducing GSH. Cu(I)‐catalyzed Fenton reactions and the H_2_O_2_ production by GOx increase ROS levels and lead to enhanced ferroptosis; another common feature between ferroptosis and cuproptosis lies in cancer metabolism. Cells with high mitochondrial activity and an altered redox balance may be particularly susceptible to both ferroptosis and cuproptosis, suggesting overlapping metabolic vulnerabilities. This crosstalk is further supported by evidence that certain ferroptosis inducers, such as erastin and sorafenib, can also promote cuproptosis [38]. This dual effect is achieved by promoting the oligomerization of Cu‐dependent lipoylated proteins by inhibiting mitochondrial proteases, which typically degrade the FDX1 protein. Moreover, ferroptosis inducers reduce GSH synthesis, exacerbating cuproptosis [38].

Other forms of cell death are also related to cuproptosis. For instance, Cu can induce autophagy via the mtROS‐dependent Akt/AMPK/mTOR signaling pathway [39]. Additionally, a Cu‐bacteriochlorin nanosheet that induces pyroptosis has shown increased tumor immunogenicity and antitumor effects in both in vivo and in vitro studies while minimizing systemic side effects [40]. Beyond cuproptosis and ferroptosis, emerging evidence suggests that metal‐dependent cell death pathways may represent a broader category of targetable vulnerabilities in cancer. Systematic exploration of how Cu, iron, and other transition metals orchestrate cell death could reveal novel therapeutic combinations and identify biomarkers for patient stratification.

## 2 Cuproptosis Inducers and Their Mechanism of Action

Cu ionophores are a class of molecules that have garnered significant attention in recent years due to their ability to increase intracellular Cu levels. They have been proposed for various biomedical applications [41]; however, here we will focus specifically on their antitumor activity.

**FIGURE 5:**
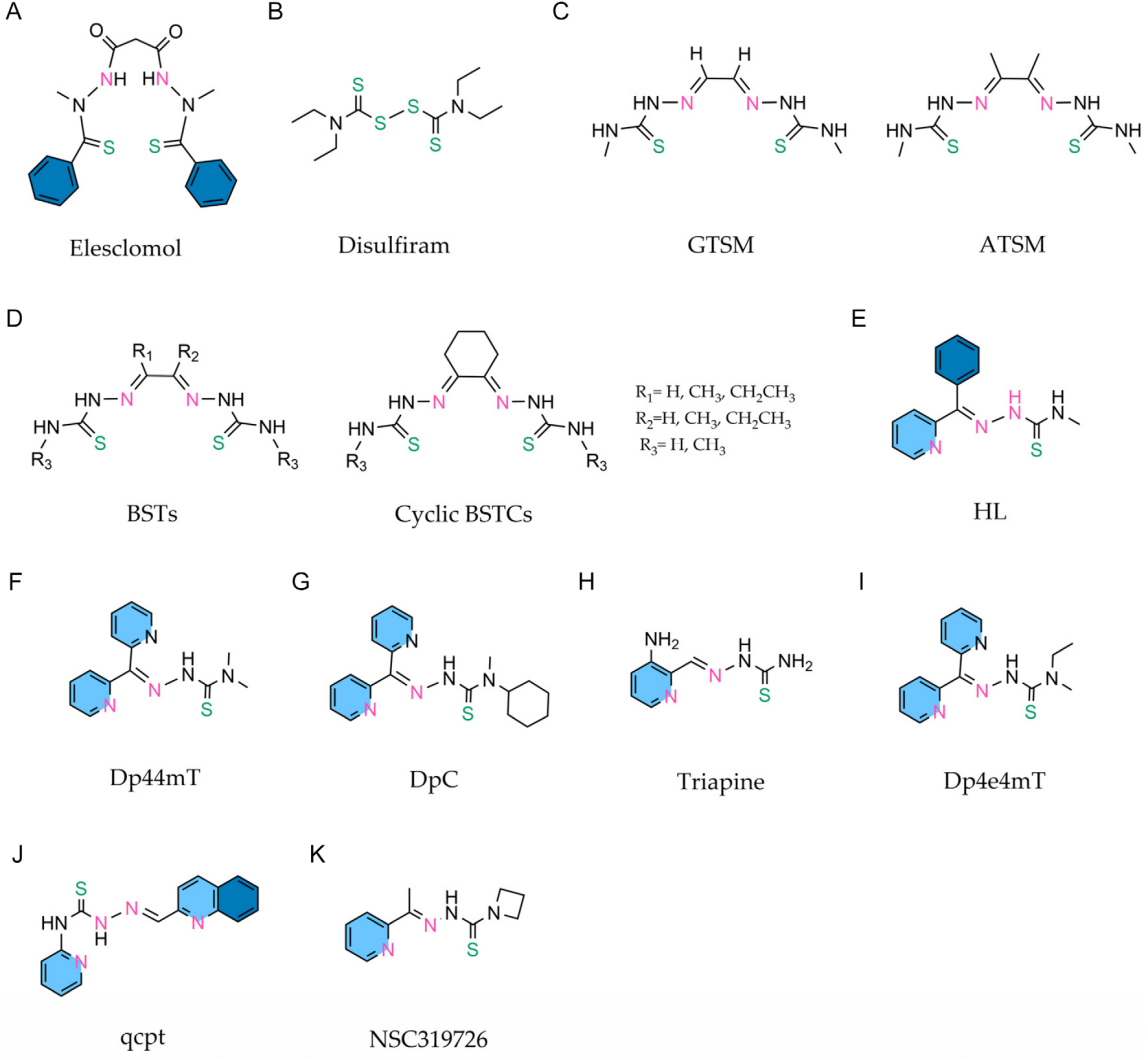
Chemical structures of some Cu ionophores: (A) elesclomol, (B) disulfiram, (C) GTSM and ATSM, (D) BSTs and cyclic BSTs, (E) HL, (F) Dp44mT, (G) DpC, (H) triapine, (I) Dp4e4mT, (J) qcpt, (K) NSC319726.

Cu ionophores can trigger cuproptosis, and some of them exhibit a slight selectivity for cancer cells over healthy ones. However, several approaches have been proposed to further improve their selectivity. A few years ago, we provided a comprehensive overview of these strategies used up to that point [42]. Research into Cu ionophores has expanded considerably between 2005 and 2025, leading to the identification and exploration of various classes of ionophores with biological applications in cancer therapy. These include dithiocarbamates, thiosemicarbazones, quinolines (HQs and AQs), flavones, etc. (Figure 5).

### 2.1 Elesclomol

ES is one of the most extensively studied Cu ionophores (Figure 5A). It forms a neutral 1:1 complex with Cu, where the metal center adopts a distorted square‐planar geometry, coordinated by sulfur and amide nitrogen atoms of the ligand [43], as confirmed by crystallographic data. Moreover, electrochemical analysis has revealed that the complex undergoes a reversible Cu(II)/Cu(I) redox transition, with reduction potentials lying within the biologically relevant electrochemical range, thereby enabling Cu redox cycling under physiological conditions [43]. The ES complex is delivered into the cell and mitochondria, where Cu ions are subsequently reduced to Cu(I) by FDX1 [44]. This process facilitates lipoylation and aggregation of mitochondrial TCA cycle enzymes, especially DLAT, and leads to the loss of Fe–S cluster proteins [45]. It also triggers ROS production, oxidative stress, and GSH depletion through the reduction of Cu and Fenton‐like reactions catalyzed by Cu^+^, causing mitochondrial dysfunction. Subsequently, ES is exported from the cell and can re‐enter, repeatedly transporting Cu into intracellular compartments [46].

Unlike other ionophores such as DSF, ES exhibits strong mitochondrial selectivity for Cu. Importantly, its activity depends specifically on Cu, as complexes with redox‐stable metals (e.g., Ni^2+^, Pt^2+^) display minimal cytotoxic effects, emphasizing Cu as the key mediator of its antitumor action [47]. The role of ES in cancer therapy is further strengthened by its ability to promote ferroptosis through multiple mechanisms, including suppression of ATP7A, ROS accumulation, degradation of SLC7A11, and disruption of iron homeostasis. At the molecular level, Cu delivered by the ES–Cu complex is also transported to the Golgi apparatus, where it supports the metalation of the multicopper oxidase Fet3 [48]. Active Fet3 oxidizes Fe(II) to Fe(III), facilitating iron uptake via transferrin receptor 1 (Ftr1), ultimately disturbing mitochondrial iron homeostasis. This multifaceted approach makes ES a powerful agent in the fight against cancer, particularly in cases where traditional therapies fall short.

### 2.2 Dithiocarbamates

This class of compounds has been extensively studied for its ability to induce cancer cell death in a panel of various tumors [49]. Dithiocarbamates coordinate with Cu due to resonance between their dithiocarbamate and thioureide forms, which delocalizes electron density toward sulfur and enhances its donor ability [50]. This allows stabilization of Cu in multiple oxidation states through strong sulfur–metal interactions. The substituents on nitrogen can further modulate the electronic properties and steric hindrance of the ligand, thereby influencing the geometry and coordination [50]. Depending on the M/L ratio and Cu oxidation state, both ML and ML_2_ species can be formed. ML_2_ species are generally more stable and typically adopt tetrahedral geometries for Cu(I) and square‐planar geometries for Cu(II) [51]. A representative molecule of this group is DSF (Figure 5B). Initially approved by the FDA in 1951 for the treatment of chronic alcohol addiction as an acetaldehyde dehydrogenase inhibitor, DSF has found new potential in oncology [52]. More recently, numerous preclinical studies have shown significant cytotoxic effects against breast cancer, melanoma, non‐small cell lung cancer (NSCLC), HCC, and prostate cancer, with this effect being markedly enhanced in the presence of Cu [53, 54].

The antineoplastic efficacy of DSF is mainly attributed to the formation of its active metabolite diethyldithiocarbamate (DTC), which binds Cu ions and generates a DTC–Cu complex [55]. This complex promotes intracellular Cu accumulation and undergoes redox cycling, thereby producing elevated levels of ROS. The resulting oxidative stress causes extensive damage to DNA, proteins, and lipids, overwhelming the antioxidant defenses of tumor cells and contributing to their death [56]. In addition, Cu‐DTC inhibits the proteasome, which leads to the accumulation of misfolded proteins and the induction of cellular stress, ultimately triggering apoptosis in malignant cells [57].

In HCC, DSF‐Cu has been demonstrated to inhibit metastasis by down‐regulating the NF‐κB and TGF‐β signaling pathways, thereby reducing the epithelial–mesenchymal transition process [58]. Moreover, DSF‐Cu disrupts mitochondrial homeostasis, increases free iron levels, and plays a crucial role in ferroptosis, a key mechanism of DSF‐Cu‐induced cell death. Several independent studies support the involvement of the DSF‐Cu complex in promoting ferroptosis [32, 33, 59].

Recently, DSF has emerged as a potent inducer of cuproptosis, emphasizing its potential as an agent capable of targeting multiple cell death pathways [60, 61, 62]. To cite a few examples, in a study by Huang et al., DSF was shown to induce cell death in vitro and in vivo in pituitary neuroendocrine tumor cells through cuproptosis [63]. The DSF‐Cu complex led to the accumulation of Cu within these cells, triggering mitochondrial dysfunction and subsequent cell death. Importantly, DSF‐Cu treatment not only reduced cell proliferation in vitro but also significantly suppressed tumor growth in vivo in xenograft models.

Remarkable outcomes have also been achieved when the compound was administered in association with other drugs. DSF‐Cu has been shown to exert anticancer effects across several tumor types, including NSCLC. Li et al. investigated the combination of DSF and anti‐PD‐L1 therapy in A549 cells, a human NSCLC cell line [64]. They found that DSF treatment increased the expression of ATP7B and PD‐L1. The upregulation of PD‐L1 favors immunosuppression and tumor immune escape, thereby limiting the therapeutic efficacy of DSF. To overcome this, the addition of anti‐PD‐L1 or other inhibitors such as JQ‐1 can significantly improve DSF antitumor activity [64]. ATP7B contributes to resistance by increasing the efflux of Cu outside the cell and activating the HIF‐1 signaling pathway, which regulates PD‐L1 expression. The use of an HIF‐1 inhibitor (PX478) in combination with DSF further promoted apoptosis and cuproptosis in NSCLC cells [64]. Nonetheless, the study has important limitations, as it was carried out in a single cell line (A549), and further validation in additional models, including animal and clinical studies, is required.

The versatility of DSF in inducing various forms of cell death is particularly promising for treating drug‐resistant cancers.

### 2.3 Thiosemicarbazones (TSCs)

Thiosemicarbazones (TSCs) are a versatile class of ligands whose donor atoms participate in bidentate coordination, forming a stable five‐ or six‐membered chelate ring with transition metals such as Cu. They are characterized by the presence of an azomethine nitrogen, a hydrazinic nitrogen, and thione–thiol tautomers, functional groups that enable binding to metals in both anionic and neutral forms [65]. Depending on their denticity and pH, these ligands typically form either ML or ML_2_ species. In particular, bis‐thiosemicarbazones (BSTCs) are tetradentate and generally coordinate Cu in a 1:1 stoichiometry with two N and two S donor sites [66]. As a result, they tend to produce stable neutral ML complexes rather than ML_2_ species.

Due to their strong metal‐binding properties, TSCs have garnered considerable attention in antitumoral research, especially for their ability to form Cu complexes with promising cytotoxic potential [67]. The anticancer activity of TSCs is closely linked to their structure, including the nature and position of substituents, which can modulate key parameters such as lipophilicity, redox potential, and Cu transport capacity.

Among them, some of the most characterized TSCs are glyoxal‐bis(N4‐methylthiosemicarbazone) (GTSM) and diacetyl‐bis(N4‐methylthiosemicarbazone) (ATSM), which contain a pair of TSC motifs and act as tetradentate ligands that form neutral, lipophilic Cu(II)L complexes (Figure 5C).

A study on a group of BSTC ligands, unsubstituted, monosubstituted, or disubstituted at the diamine positions, demonstrated that the structural variation significantly impacts their Cu(II)/Cu(I) redox potentials, lipophilicity, and antiproliferative activity. Unsubstituted and monosubstituted ligands (Figure 5D) exhibited greater hydrophilicity, less negative Cu(II)/Cu(I) redox potentials, and significant antineoplastic activity against SK‐N‐MC neuroepithelioma cells, with IC_50_ values in the low nanomolar range [68]. These were significantly more active than deferoxamine (DFO) and triapine (3‐AP), used as references. Moreover, complexation with Cu did not markedly alter their antiproliferative properties, with Cu complexes showing activity comparable to the free ligands.

In contrast, disubstituted bis(thiosemicarbazones) (Figure 5D) exhibited increased hydrophobicity, more negative Cu(II)/Cu(I) redox potentials, and lower cytotoxicity, displaying a pronounced enhancement in antiproliferative effects, more than a sixfold increase compared to the corresponding free ligands.

Stefani et al. demonstrated that the cytotoxicity of these compounds is mediated by the intracellular accumulation of Cu and the generation of ROS [68]. Moreover, with the use of lysosomotropic fluorescent probes, the study found that GTSM–Cu complex, once internalized, is preferentially localized to the lysosomes, where redox cycling initiates oxidative stress, leading to the disruption of lysosomal membrane integrity and subsequent cell death.

Another compelling example of a system demonstrating noteworthy antineoplastic activity is the thiosemicarbazone–Cu complex [Cu(II)_2_Cu^I^(L)_2_Cl_3_], where HL is the tridentate (E)‐N‐methyl‐2‐(phenyl(pyridin‐2‐yl)methylene) hydrazinecarbothioamide ligand. This complex displayed stronger anticancer activity than cisplatin, with comparable hemolytic effects (Figure 5E) [69]. In three‐dimensional A549 tumor spheroid models, the complex markedly suppressed tumor growth, induced extensive cell death, and inhibited A549 tumor growth in a xenograft mouse model with minimal systemic toxicity. Regarding the mechanism of action, proteomic and functional analyses revealed that the complex primarily disrupts mitochondrial bioenergetics, affecting the TCA cycle, oxidative phosphorylation, and respiratory chain activity, thereby reducing oxygen consumption. Mechanistically, the complex activates multiple cell death pathways, including cuproptosis (DLAT aggregation and FDX1 downregulation), autophagy/mitophagy (PINK1–Parkin pathway and LC3B‐II accumulation), and apoptosis (cytochrome c release, caspase‐3 activation, and ROS generation).

Among TSCs, di‐2‐pyridylketone 4,4‐dimethyl‐3‐thiosemicarbazone (Dp44mT) (Figure 5F) and its optimized analog di‐2‐pyridylketone 4‐methyl‐4‐cyclohexyl‐3‐thiosemicarbazone (Dpc) (Figure 5G) are often taken as reference compounds due to their high cytotoxic potency, well‐characterized redox mechanisms, and promising efficacy, especially when combined with other antitumoral drugs [70, 71, 72].

3‐AP, an α‐pyridyl thiosemicarbazone (Figure 5H), is currently in clinical trials for the treatment of glioblastoma (GBM) [73, 74] and vaginal and cervical cancers [75, 76]. It shares the same metal‐binding scaffold as Dp44mT but displays markedly lower potency, being ∼100‐fold less cytotoxic [77]. Notably, while 3‐AP is inactivated by Cu in a 1:1 ratio, Cu potentiates Dp44mT cytotoxicity in a dose‐dependent manner. The difference in potency between 3‐AP and Dp44mT can be attributed to the fact that only Dp44mT functions as a Cu ionophore, which enhances its cytotoxicity. Consequently, despite the structural similarity of the two compounds, they exhibit distinct mechanisms of cytotoxicity: 3‐AP primarily acts through ribonucleotide reductase inhibition and DNA synthesis arrest, whereas Dp44mT induces rapid cell death modulated by Cu [78]. However, no studies have specifically demonstrated that Dp44mT induces cuproptosis, as this cell death mechanism was identified after the initial investigations of Dp44mT. Although Dp44mT effectively inhibits tumor growth and overcomes drug resistance, its clinical potential is limited by cardiotoxicity at higher doses [78]. To address this, DpC was developed, showing greater anticancer efficacy and significantly improved tolerability.

Dp44mT and DpC have been shown to overcome P‐glycoprotein (Pgp)‐mediated drug resistance by exploiting a lysosome‐targeting mechanism, demonstrated in both in vitro and in vivo models [79]. Pgp is a membrane transporter that actively pumps drugs out of cells using ATP. Its overexpression in cancer cells leads to Pgp‐mediated drug resistance, reducing the effectiveness of chemotherapy. This mechanism of resistance is based on the ionization of Dp44mT and DpC, which become protonated within the acidic lysosomal environment, leading to their selective accumulation in this organelle, a key site of action for these compounds. Following the degradation of iron‐ and Cu‐binding proteins in lysosomes, the released metal ions are chelated by Dp44mT and DpC, generating redox‐active complexes. These complexes promote oxidative damage to the lysosomal membrane, ultimately triggering cell death in cancer cells [70].

Notable for their activity, compounds belonging to this class, for which a cuproptotic mechanism has not been stated but another type of mechanism has, are reported by Dharmasivam et al. and Hu et al.

Dharmasivam et al. highlight the strong antiproliferative and redox activity of a new TSC derivative, [Cu(Dp4e4mT)Cl] (Figure 5I), which does not undergo transmetallation with Fe(III), thereby avoiding harmful oxidation of oxy‐myoglobin and oxy‐hemoglobin [80]. Importantly, this Cu complex is internalized into tumor cells, accumulates in lysosomes, and remains redox‐active, generating ROS that trigger lysosomal damage and tumor cell death. These findings, combined with its high cytotoxicity demonstrated in SK‐N‐MC neuroepithelioma cells and AsPC‐1 pancreatic cancer cells, support its potential as a promising anticancer agent.

Hu et al. have rationally designed two Cu complexes obtained from quinoline–thiosemicarbazone conjugate: [Cu(qcpt)(PPh_3_)_2_Br]·2CH_3_CN (**1**) and [Cu_2_(qcapt)(Ac)_2_(CH_3_O)] (**2**), where qcpt = quinoline‐2‐carboxaldehyde‐4‐pyridine‐3‐thiosemicarbazone and qcapt = quinoline‐2‐carboxaldehyde acid‐4‐pyridine‐3‐thiosemicarbazone (Figure 5J) [81]. The ligand provides a quinoline and pyridyl nitrogen donor atom, along with the thiosemicarbazone sulfur, to coordinate with Cu. X‐ray crystallography reveals that complex **1** is tetra‐coordinated with Cu(I), involving two PPh_3_ ligands, one bromine atom, and one sulfur atom, arranged in a trigonal pyramidal geometry. In complex **2**, one Cu center coordinates with two nitrogen atoms, one sulfur atom, and one carboxylate oxygen atom, adopting a square planar geometry, while the second Cu center coordinates with two nitrogen atoms and three oxygen atoms, resulting in a square pyramidal geometry. In vitro antitumor studies showed that these complexes exhibited effective antiproliferative activity against the malignant tumor cell lines HCT116 and SMMC7721 (human colon and liver carcinoma cells, respectively), with complex 1 being particularly effective. Furthermore, mechanistic studies revealed that complex 1 readily enters cells, accumulates in mitochondria and nuclei, blocks the S‐phase of the cell cycle, induces mitochondrial dysfunction and ATP depletion, increases ROS levels, and ultimately triggers cell death. Thus, complex 1, with its efficient cellular uptake and dual DNA and mitochondrial targeting, shows promise as a candidate for anticancer therapy.

Among TSCs, NSC319726 (NSC, Figure 5K) has also been indicated as another Cu ionophore with significant anticancer potential [82]. NSC exerts potent cytotoxicity in GBM cells by binding Cu, which induces oxidative stress, DNA damage, and G1 cell‐cycle arrest even at very low concentrations. Unlike its reported zinc ionophore activity, this effect is not dependent on zinc. Pharmacogenomic and supplementation studies confirmed a Cu‐mediated mechanism, while hypoxia via HIF‐1α significantly reduced this toxicity. Overall, the activity of NSC results from Cu dysregulation and oxidative DNA damage.

### 2.4 Quinolines (HQs and AQs)

**FIGURE 6:**
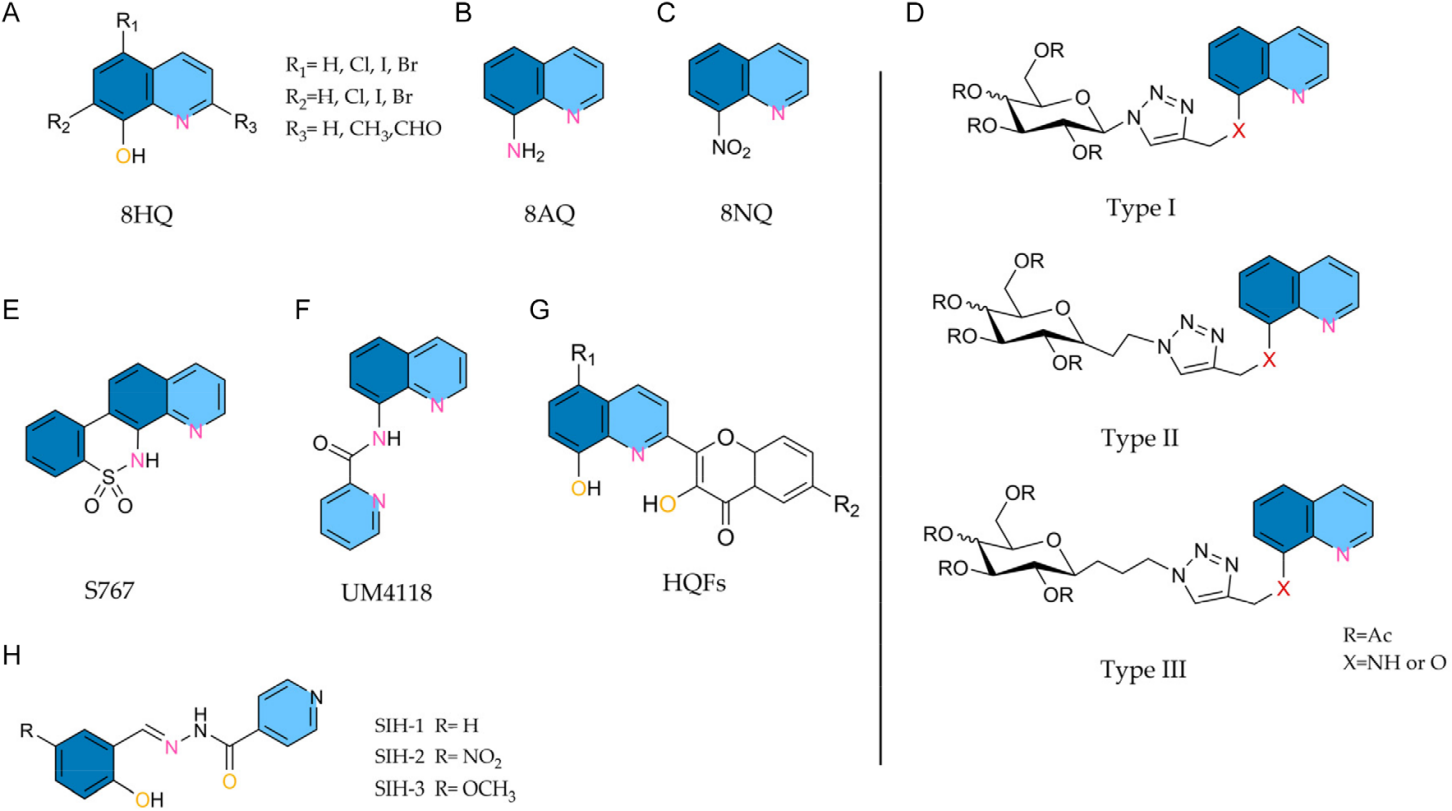
Chemical structures of quinolines and hydrazones: (A) 8HQ, (B) 8AQ, (C) 8NQ (D) glycoconjugates of quinolines Type I, II, and III, (E) S767, (F) UM4118, (G) HQFs, (H) SIH‐1 and its derivatives (SIH‐2 and SIH‐3).

8‐Hydroxyquinolines and 8‐aminoquinolines (Figure 6A,B, 8HQs and 8AQs) are bidentate ligands with a bicyclic heterocyclic structure. Both coordinate Cu similarly, forming ML and ML_2_ species and rings with the same number of atoms. The only difference is that 8AQ has two nitrogen donor atoms, the pyridine nitrogen and the amino group at position 8, while 8HQ coordinates through nitrogen and oxygen donor atoms. This results in the binding constants generally being higher for 8HQ than for its 8AQ analogs.

A study by George and coworkers presents a detailed structural investigation of Cu(II) complexes formed with selected 8HQs in aqueous solution using synchrotron XAS, HERFD‐XAS, EPR, and DFT calculations [83]. This work provides insights to connect in vitro structural information with potential in cell/*vivo* behavior. It was found that 5,7‐dihalogenated 8HQ derivatives coordinate Cu(II) in a pseudo square planar geometry with two bidentate ligands, with planarity and symmetry confirmed by EXAFS analyses and multiple scattering effects involving halogen substituents. Substituents in the 2‐position, such as methyl or aldehyde groups, were found to distort this planarity, resulting in a “propeller‐like” arrangement around the metal center. These distortions arise from both steric and electronic effects and are partially stabilized by hydrogen bonding between the ligands. The resulting complexes exhibit reduced LMCT transitions and dampened EXAFS oscillations, reflecting the deviation from coplanarity. These structural findings carry important implications for drug design. Substituents at positions 5 and 7 can modulate hydrophobicity, potentially influencing membrane permeability and cellular trafficking, whereas 2‐position substituents affect the Cu(II) coordination environment, planarity, and overall geometry of the complexes. Such knowledge could be used for the development of 8HQ‐based therapeutics capable of crossing in vivo barriers and targeting metal ion homeostasis in diseases associated with Cu trafficking and storage [84]. For several 8HQs and 8AQ, an ionophoric‐type mechanism has been demonstrated or hypothesized [85, 86, 87, 88, 89, 90]. The presence of Cu ions, whether the complex is synthesized beforehand or generated in situ, significantly enhances the activity of many 8HQs as well as 8AQ, underscoring the critical importance of the complex formation [84, 91, 92, 93].

In a review we published a few years ago, we provided a comprehensive summary of HQs used as anticancer agents in combination with Cu and detailed the various targets reported for these systems [41, 42]. All these studies were conducted before the discovery of cuproptosis. Here, we restrict our discussion to 8AQ articles and a limited number of 8HQ studies on anticancer activity that have appeared since that review.

As for 8AQ, it exhibited negligible activity against the cancer cell lines A549, HepG2, and HCT116 when administered alone, whereas its combination with Cu markedly enhanced cytotoxicity, nearly doubling its antiproliferative effect. Supporting the critical role of Cu binding, the 8‐nitroquinoline (8NQ) derivative (Figure 6C) showed no significant antiproliferative activity under identical conditions and no appreciable enhancement in the presence of Cu. The authors further hypothesize, based on a series of in vitro studies, that upon entering tumor cells, the 8AQ–Cu complex interacts with intracellular GSH, promoting complex dissociation and Cu release. This process underlies the proposed ionophoric mechanism of 8AQ, enabling intracellular Cu accumulation and subsequent overload. The involvement of intracellular reducing agents, such as GSH, is therefore crucial, as they act as triggers for Cu release within cells. The biological relevance of these findings was further supported by an in vivo study using zebrafish embryos [93].

Pastuch‐Gawołek and Szreder explored whether replacing the hydroxyl group of 8HQ with an amino group could improve the tumor selectivity of glycoconjugate analogs (Figure 6D) [94]. To test this, they synthesized glucose‐ and galactose‐conjugated 8AQ derivatives via Cu(I)‐catalyzed azide–alkyne cycloaddition, using triazole linkers with alkyl chains of different lengths. The structural modification at the donor site altered the metal binding profile, and the MTT assays conducted on cancer cell lines HCT‐116 and MCF‐7 demonstrated that two glycoconjugates (compounds 17 and 18) showed superior potency and selectivity toward tumor cells compared to their 8HQ analogs. Further experiments carried out in media containing nontoxic concentrations of Cu(II) revealed a significant increase or onset of bioactivity in the 8AQ glycoconjugates. This finding suggests that the antitumor activity of these derivatives depends on Cu coordination [94].

Within the class of aminoquinolines, S767 and UM4118 can also be included (Figure 6E,F). In particular, UM4118 was developed based on SAR studies aimed at optimizing the compound S767 [95]. Unlike other ionophores such as clioquinol (CQ), inductively coupled plasma (ICP)‐MS analysis showed that UM4118 is a selective Cu ionophore as it selectively increases intracellular Cu without significantly affecting iron or zinc. Unlike S767 and its sulfonamide analogs, which induce significant DNA damage (γH2AX), UM4118 is nongenotoxic. Furthermore, cell studies identified UM4118 as a more potent compound (26‐fold increase in IC50) in acute myeloid leukemia OCI‐AML5 cells. A series of experiments conducted in the presence and absence of Cu, along with the analysis of specific markers, fully demonstrated that UM4118 behaves like other known ionophores, inducing cuproptosis. AML cells harboring SF3B1 mutations (often associated with a poor prognosis) or exhibiting ISC deficiencies (lack or dysfunction of ISC proteins) are susceptible to compounds such as UM4118 that trigger cuproptosis. This selective vulnerability not only underscores a novel mechanism of action but also opens promising avenues for the development of targeted and potentially more effective therapeutic strategies in AML and related malignancies.

Recently, a new class of pseudonatural flavonols bearing HQ (HQFs, Figure 6G) has been developed by replacing the B‐ring of 3‐hydroxyflavone (3‐HF) with 8HQ, thereby combining flavonoid bioactivity with Cu ionophoric properties [96]. In particular, the formation constants (log K) of these Cu(II)–HQF complexes range from approximately 10 to 11.3, indicating the formation of stable 1:2 metal–ligand species, where Cu is coordinated by two hydroxyl oxygen atoms together with a nitrogen donor from the HQ moiety. The Cu‐ionophoric ability of HQFs was demonstrated in HeLa cells using fluorescence imaging and ICP‐MS. HQFs performed comparably to the well‐established ionophores such as ES in facilitating cellular Cu uptake. The intracellular Cu accumulation promoted by HQFs could trigger cuproptosis. In biological assays, the free ionophores HQF, HQF‐Cl‐1, and HQF‐OMe showed no intrinsic toxicity, whereas when administered with Cu, they displayed strong selective cytotoxicity, effectively killing HepG2 liver cancer cells while sparing normal primary mouse hepatocytes [96]. This selectivity highlights the greater sensitivity of malignant cells to Cu overload and their predisposition to undergo Cu‐dependent cell death.

### 2.5 Hydrazones

Salicylaldehyde isonicotinoyl hydrazone (SIH‐1, Figure 6H) belongs to this class of compounds and is a pyridoxal isonicotinoyl hydrazone analog originally designed as an iron chelator to exploit the elevated iron demand of cancer cells. Besides Fe(III), SIH‐1 can also coordinate Cu as a monobasic tridentate ligand (through the phenolic oxygen, carbonyl oxygen, and azomethine nitrogen), forming a stable 1:1 Cu(II) complex. The Cu ionophoric behavior was demonstrated by Zhou's group [97]. Thanks to the excellent scaffold for structural tuning, a series of SAR studies have been conducted. The importance of the phenolic hydroxyl group for Cu coordination and, consequently, for the ionophoric activity was demonstrated by its methylation, which significantly reduced Cu binding. Furthermore, the introduction of electron‐withdrawing and electron‐donating groups at the *para*‐position of the phenolic hydroxyl (in SIH‐2 and SIH‐3, respectively, Figure 6H) resulted in reduced cytotoxic activity compared to SIH‐1. Among the series, SIH‐1 proved to be the most efficient compound against HepG2 cancer cells, while showing remarkable selectivity over normal HUVEC cells. Collectively, these derivatives allowed a systematic investigation of how electronic modifications govern Cu(II) transport and anticancer efficacy, paving the way for the rational design of Cu(II)‐based ionophoric anticancer agents (PAAs) [97].

Another study has reported on the effects of alkyl chain length on Cu transport and anticancer activity [98]. In brief, a series of Cu ionophores (C2–C10) was synthesized through n‐alkyl modification of a Schiff base derivative that shares the metal‐binding motif with SIH‐1. The antiproliferative effects of these compounds were assessed against TNBC cell lines, including murine 4T1 and human MDA‐MB‐231 cells, using the MTT assay, with cisplatin and SIH‐1 as reference drugs. The results showed that antiproliferative efficacy followed the trend C6 > C8 > C10 > C4 > C2, with C6 being the most active compound, regardless of Cu supplementation. The addition of Cu ions markedly enhanced activity, especially for C6, whose antitumor efficacy increased more than tenfold, overcoming SIH‐1 in 4T1 and MDA‐MB‐231 cells under the same conditions. Without Cu supplementation, the IC_50_ values of C6 and SIH‐1 in 4T1 cells were 5.04 and 18.04 μM, respectively, and in MDA‐MB‐231 cells, they were 14.42 and 19.03 μM. Upon the addition of CuCl_2_ (10 μM), the IC_50_ values of C6 dropped dramatically to 0.45 (4T1) and 1.21 μM (MDA‐MB‐231), indicating a strong synergistic effect with Cu and suggesting a mechanism of action as an ionophore. The superior performance of C6 is attributed to its balanced lipophilicity and optimal Cu‐binding affinity, which facilitate efficient metal transport. Mechanistically, ionophore‐mediated Cu transport involves partitioning into the membrane, translocation across the lipid bilayer, and intracellular ion release. Molecules that are too hydrophilic or too hydrophobic exhibit poor translocation efficiency. Finally, it has been demonstrated that C6 promotes cuproptosis. In vivo studies further demonstrated that C6 not only exhibits strong tumor inhibition but also exertes immunomodulatory effects. This study provides valuable insights into designing new systems that can target metal homeostasis.

### 2.6 Natural Cu Ionophores

**FIGURE 7:**
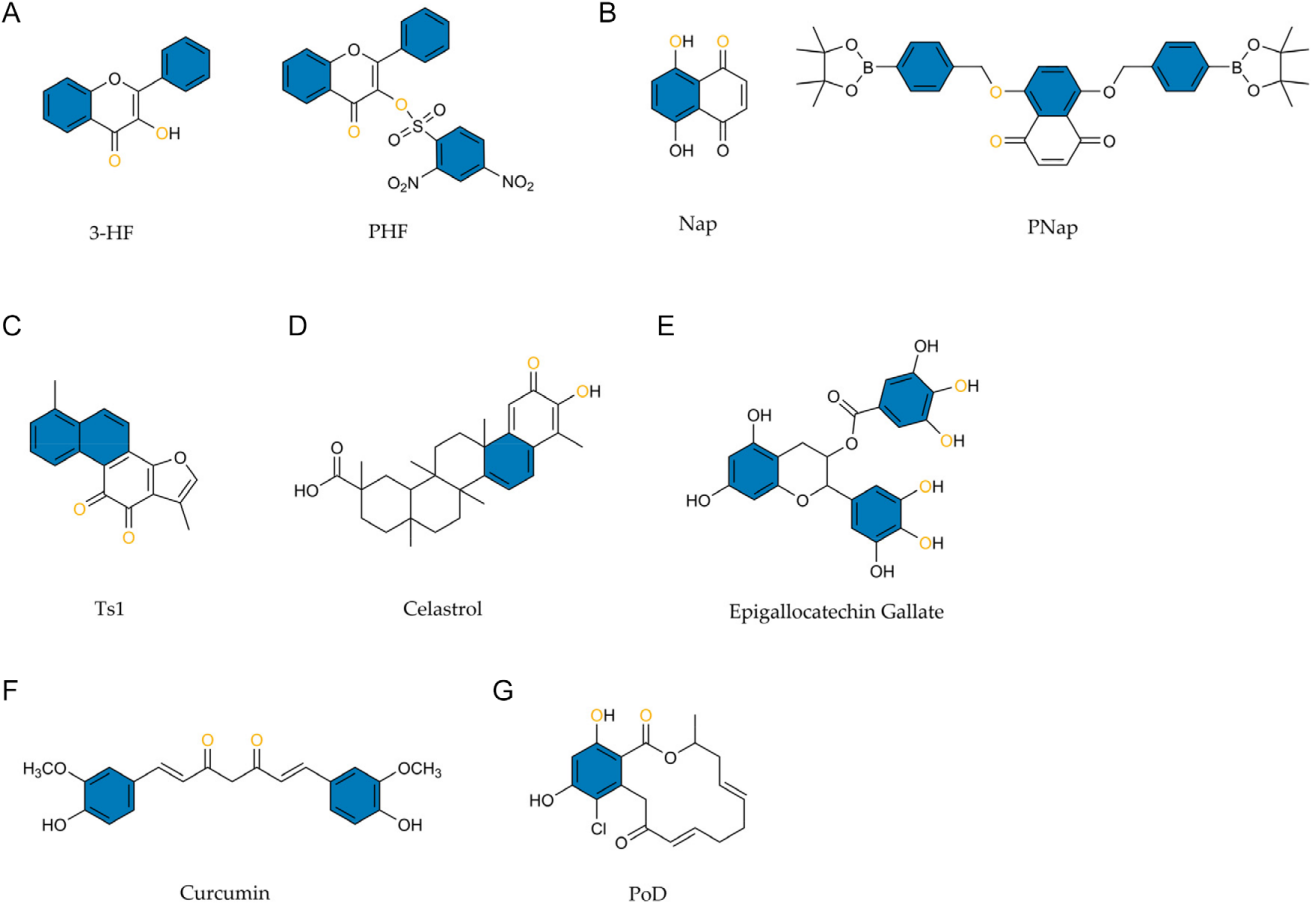
Chemical structures of natural Cu ionophores: (A) 3‐HF and its derivative PHF, (B) Nap and its derivative PNap, (C) Ts1, (D) celastrol, (E) epigallocatechin gallate, (F) curcumin, (G) PoD.

Natural compounds, including flavonoids and curcumin derivatives, are emerging as promising candidates for Cu ionophore activity. A series of studies by Zhou and colleagues has delineated how natural scaffolds and their derivatives can act as effective Cu ionophores, capable of selectively inducing oxidative stress in cancer cells. One such compound, 3‐HF (Figure 7A), can bind Cu(II) and form a neutral, lipophilic complex (ML_2_) due to its 3‐hydroxy‐4‐keto group. 3‐HF‐Cu can efficiently cross cell membranes. Once internalized, the reducing environment of the cytoplasm, particularly the presence of GSH, promotes the dissociation of the 3‐HF‐Cu complex. While the initial Cu release due to GSH is stoichiometric, the subsequent Cu(I)‐mediated redox cycling catalyzes ROS production and amplifies it. It amplifies GSH depletion, ultimately leading to cell death, as demonstrated in HepG2 liver cancer cells [99]. A protected proionophore derivative (PHF, Figure 7A) was synthesized by masking the enolic hydroxyl with a protecting thiol‐sensitive group [100]. PHF is susceptible to nucleophilic attack by GSH, leading to a more selective deprotection in GSH‐rich cancer cells and minimizing the activation in normal cells due to their lower GSH levels. With a similar approach, the team developed a boronate‐protected naphthol derivative (PNap) from naphthazarin (5,8‐dihydroxy‐1,4‐naphthoquinone, Nap), a compound extracted from the roots of *Lithospermum erythrorhizon* (Figure 7B) [101]. The structure of Nap features donor atoms analogous to those in 3‐HF, suggesting potential activity as a Cu(II) ionophore. PNap is activated in cancer cells via hydrogen peroxide‐triggered deprotection, releasing Nap and a quinone methide, both capable of GSH depletion through alkylation [102]. This alkylation also facilitates the efflux of Nap–GSH adduct from the cells. Then, as observed with 3‐HF, the resulting Nap–GSH adduct may act as a Cu ionophore, perpetuating oxidative damage.

Most recently, Zhou's group identified tanshinone I (Ts1, Figure 7C), a natural o‐quinone, as a novel Cu ionophore [103]. Cu induces the tautomerization of Ts1 from its diketo to keto‐enol forms, promoting a deprotonated bidentate site involving the o‐quinone oxygen atoms. Ts1 coordinates Cu in a 2:1 ligand‐to‐metal stoichiometry, forming a neutral, lipophilic Cu complex (ML_2_), which can cross cell membranes. In vitro, the complex preferentially affects cancer cells (e.g., HepG2) over normal ones, likely due to their higher intracellular levels of reducing agents like GSH, essential for the Cu release from Cu/Ts1 complex. The resulting cellular Cu accumulation, disruption of redox homeostasis, and ROS production drive cuproptosis.

Curcumin (CUR, Figure 7F), extracted from *Curcuma longa*, has been identified as a potential Cu ionophore capable of modulating cuproptotic pathways. Recent computational and experimental studies on CUR–Cu coordination chemistry have highlighted that this natural molecule, through its β‐diketone moiety, forms both 1:1 and 1:2 metal–ligand complexes, improving its solubility, stability, and antioxidant efficacy compared to free CUR [104]. The single‐crystal X‐ray structure of the homoleptic Cu–CUR complex (ML_2_) evidences a square‐planar geometry around Cu, with the β‐diketone chelating the central metal ion, reinforced by C–H···O supramolecular interactions and methanol‐mediated crystal packing [105]. This complex not only exhibited enhanced aqueous solubility (log *P* ≈ 2.3) but also demonstrated superior cytotoxicity against several cancer cell lines [105].

Based on quantitative proteomic analysis, CUR–Cu complex promotes Cu uptake and its accumulation inside the cell [106]. Moreover, CUR modulates critical metabolic pathways in colorectal cancer cells. Particularly, those related to lipid, RNA, and NADH/NADPH metabolism. At the same time, it upregulates positive mediators of cuproptosis, including MRPS14, GCLM, IMP4, and FAU, which enhance Cu‐dependent cell death in excess of the normal Cu levels [106].

In a recent work, Li Feng et al. isolated 24 resorcylic acid lactones (RALs) from the fungus *Ilyonectria* sp. A structure–activity relationship (SAR) analysis revealed that the pharmacophore structure responsible for their antitumoral activity is the α,β‐unsaturated ketone moiety, particularly when located at the C8–C10 position. Biological potency was further enhanced by the presence of an epoxide and a C4–C5 double bond, whereas removal of the chlorine atom at C13 or the introduction of a C6–C7 double bond significantly reduced efficacy. Among the isolated RALs, pochonin D (PoD, Figure 7G) exhibited potent antiproliferative effects against TNBC cells, both in vitro and in vivo, with less toxicity toward normal breast cell lines, including MCF10A [107]. Mechanistic investigations demonstrated that PoD acts as a Cu ionophore, triggering cuproptosis in TNBC cells by promoting intracellular Cu accumulation. Furthermore, the study identified peroxiredoxin 1 (PRDX1) as a potential biomarker associated with cuproptosis and a therapeutic target in TNBC. PoD was found to bind the Cys173 residue of PRDX1, suppressing its antioxidant enzymatic function and thereby inducing cuproptosis in TNBC cells, highlighting its potential as a pharmacological lead.

Collectively, these works not only reveal a common mechanism, the induction of cuproptosis based on Cu binding, intracellular accumulation, GSH‐mediated Cu release, redox imbalance, and ROS‐induced cell death, but also underscore the intrinsic Cu(II) ionophoric activity of these natural or nature‐inspired molecules. This emerging property, previously unrecognized in these scaffolds, opens up new avenues for the design of pro‐oxidative anticancer agents that selectively exploit the redox sensitivity and high levels of Cu and GSH in cancer cells.

Among natural flavonoids, Celastrol (Cel, Figure 7D) and epigallocatechin gallate (EGCG, Figure 7E) do not appear to function as bona fide Cu ionophores; they nevertheless function as effective inducers of cuproptosis. Cel, extracted from the root of the *Tripterygium wilfordii* plant, has been shown to induce cuproptosis in NSCLC cells. In this research, TTM successfully countered Cel‐induced cytotoxicity, confirming the involvement of Cu in the process. Transcriptomic analysis demonstrated that Cel increases the expression of the Cu transporter CTR1, resulting in higher intracellular Cu levels, GSH depletion, and typical features of cuproptosis such as loss of iron–sulfur cluster proteins (FDX1, SDHB, POLD1), increased HSP70 levels, and DLAT oligomerization. Cel also elevated ROS levels, lowered mitochondrial membrane potential, and reduced ATP levels, further indicating mitochondrial dysfunction. In vivo, Cel significantly suppressed tumor growth without harming major organs. Overall, the findings suggest that Cel disrupts Cu balance through the SRF/CTR1 pathway, inducing cuproptosis in NSCLC cells and showing promise as a safe, effective chemotherapeutic agent [108].

Both in vitro and in vivo, EGCG significantly increased tumor cell susceptibility to Cu delivered through ES, HQ, and DSF, enhancing intracellular Cu accumulation and activating cuproptosis markers in HCC cells [109]. The mechanism involved downregulation of the axis MTF1/ATP7B, key regulators of the Cu export. However, as the authors themselves point out, the low oral bioavailability of EGCG remains restrictive, and the study should be extended to additional cell lines in order to better assess its broader applicability. Thus, the findings indicate that EGCG could represent a promising sensitizer for cuproptosis. It must be borne in mind that combinatorial therapies are generally considered particularly promising, as they offer an effective strategy to overcome the intrinsic resistance of cancer cells to individual drugs.

### 2.7 Non‐Classical Copper Ionophores

Besides the well‐known families of Cu ionophores, there are structurally different compounds that demonstrate pronounced effects on Cu transport and cell activity.

**FIGURE 8:**
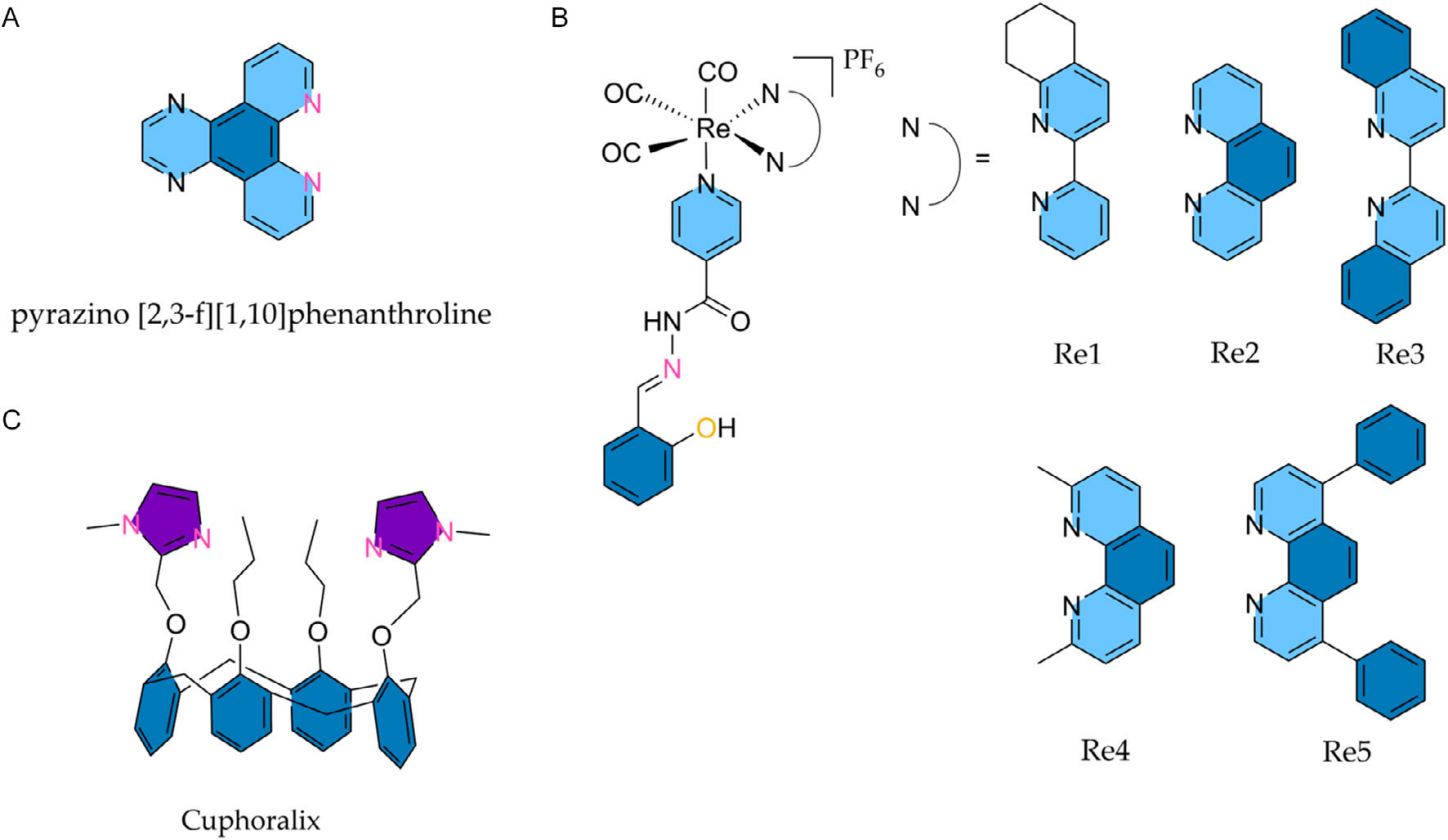
Chemical structures of nonclassical Cu ionophores: (A) pyrazino [2,3‐f][1,10]phenanthroline, (B) rhenium complexes (Re1‐Re5), (C) cuphoralix.

Li et al. reported the synthesis and characterization of four Cu complexes with pyrazino [2,3‐f][1,10]phenanthroline as the ligand (Figure 8A). Biological assays demonstrated that these complexes effectively inhibit the proliferation of TNBC cells. X‐ray crystallography revealed that the most active complex exhibits a 1:1 stoichiometric ratio of Cu to ligand and that the Cu center adopts a square‐pyramidal geometry, with a water oxygen atom occupying the apical site [110]. Mechanistically, they induce cuproptosis, as confirmed by various assays. Western blot analysis revealed downregulation of key components of the TCA cycle, such as DLAT, and upregulation of Cu transporters, including ATP7A and ATP7B, indicating cuproptosis [110]. Moreover, these complexes promote immunogenic cell death (ICD), characterized by the release of damage‐associated molecular patterns (DAMPs). These findings suggest that Cu‐complexes are promising candidates for targeted cancer therapy, leveraging both direct cytotoxicity and immune system activation.

A series of phosphorescent rhenium(I) complexes (Re1–Re5, Figure 8B) was designed to act as Cu ionophores, specifically targeting subcellular organelles to induce cuproptosis in cancer cells [111]. The geometry around the rhenium center is octahedral, characterized by a fac‐[Re(CO)_3_] core, where the metal is coordinated to a bidentate ligand and a Schiff base Cu ionophore (SIH‐1). These complexes demonstrate the ability to form a reversible 1:1 complex with Cu. In particular, the complex Re5 with a 4,7‐diphenyl‐1,10‐phenanthroline ligand shows high selectivity towards Cu(II) over other biological ions. The antiproliferative effects of Re1–Re5 were assessed in MDA‐MB‐231 and murine breast cancer cells (4T1), both with and without the addition of Cu. Among them, Re5 displayed the most favorable photophysical behavior and potent cytotoxicity. Imaging studies revealed localization of Re5 within mitochondria and the Golgi apparatus. At the same time, Western blot analysis further demonstrated that treatment with Re5 and CuCl_2_ downregulated proteins involved in the cuproptosis, such as DLAT, FDX1, and LIAS. Notably, comparable effects were also observed with Re5 alone, suggesting its ability to trigger cuproptosis through endogenous Cu.

Researchers created a family of lipophilic compounds, including cuphoralix (Figure 8C), featuring two preorganized coordinating (benz)imidazole groups specifically designed to bind Cu(I) in a linear geometry, allowing these molecules to function as ionophores, transporters that can shuttle Cu ions across cellular membranes [112]. The initial proof‐of‐concept was demonstrated in liposomes using an encapsulated Cu(I)‐sensitive fluorescent probe, confirming that these compounds could transport Cu across membranes. Importantly, six of these ionophores also restored growth in yeast cells lacking the Ctr1 transporter, demonstrating their ability to transport Cu into cells in a biologically relevant context. A key finding was that lipophilicity played a critical role in cellular activity. The four most active ionophores tested in hepatocarcinoma cells shared a relatively narrow lipophilicity range (cLogP = 7–9.2), underscoring that this molecular property is essential for effective anticancer activity [112]. What distinguishes Cuphoralix from simple Cu supplementation is its subcellular mechanism of action. Rather than simply increasing total intracellular Cu levels, Cuphoralix alters the distribution of Cu within cells by redistributing it from vesicles to the cytosol. This targeted redistribution of Cu to the cytosolic compartment creates localized metal‐induced stress, which appears to be the primary driver of its cytotoxic effects. Cuphoralix demonstrated a potent cytostatic effect across a comprehensive panel of 60 cancer cell lines [113]. In particular, in hepatocarcinoma cells, Cuphoralix exhibited anticancer activity with IC_50_ values ranging from 3 to 5 μM. Mechanistic studies revealed that Cuphoralix operates through multiple mechanistic pathways, including cuproptosis along with autophagy. The anticancer efficacy was corroborated in vivo using nude mice bearing subcutaneous A549 lung cancer xenografts, where Cuphoralix treatment, both alone and in combination with other treatments, reduced tumor volumes, suggesting potential for combination therapies. The capacity of Cuphoralix to initiate an early and irreversible cell cycle blockade while modulating the expression of key proteins involved in proliferation distinguishes it from conventional chemotherapies that primarily rely on DNA damage or apoptosis induction. This mechanistic novelty, combined with the evidence for in vivo efficacy and tolerability, positions Cuphoralix as a promising candidate for further clinical development.

Among the low‐molecular‐ weight inducers of cuproptosis, a Cu(I) complex featuring a bromine atom and three coordinated PPh_3_ groups has been recently reported [114]. The PPh_3_ group, widely used for mitochondrial targeting, was selected to facilitate the transport of Cu(I) into the mitochondria of cancer cells. Although the mechanism of this complex differs from that of ES, potentially due to the direct delivery of Cu(I) instead of Cu(II) as with ES, it nonetheless appears to activate a cell death pathway with features characteristic of cuproptosis. Specifically, treatment with the complex resulted in (1) reduced FDX1 levels, leading to decreased lipoylation of downstream proteins; (2) increased aggregation of lipoylated proteins; (3) loss of Fe–S cluster proteins; and (4) elevated HSP70 levels. Taken together, these findings position CBP as a non‐classical Cu ionophore whose biological activity extends beyond simple Cu shuttling. By directly delivering Cu(I) to mitochondria through a triphenylphosphine‐guided targeting strategy, CBP integrates redox‐driven oxidative stress with FDX1‐associated proteotoxic signaling, partially recapitulating key hallmarks of cuproptosis while diverging from the canonical mechanism described for ES.

Collectively, these systems demonstrate the increasing diversity of (nonclassical) Cu ionophores, where factors like metal oxidation state, coordination environment, and organelle‐targeting methods are intentionally designed to influence intracellular Cu transport. Instead of just serving as passive Cu carriers, these complexes function as purpose‐built metallodrugs that can alter mitochondrial Cu balance and induce cuproptosis‐related phenotypes through distinct yet convergent mechanisms. More broadly, these examples underscore a conceptual shift from simple Cu ionophores toward structurally defined Cu delivery platforms, where coordination chemistry is strategically exploited to control redox behavior, subcellular localization, and biological outcome. This emerging paradigm expands the landscape of Cu‐based anticancer strategies beyond traditional agents like ES and sets the stage for advanced Cu systems, including nanoscale platforms designed to further refine Cu speciation, targeting accuracy, and treatment selectivity.

## 3 Copper Delivery Nanoplatforms

The therapeutic application of low‐molecular‐weight Cu ionophores often remains restricted due to poor bioavailability, limited selectivity, rapid clearance from the bloodstream, and insufficient concentration of endogenous Cu [42, 115]. By way of example, ES is rapidly eliminated from plasma, with a mean half‐life of 0.79–1.06 h [116], whereas CQ, which has a half‐life between 10 and 14 h in humans, caused so many adverse effects that it was withdrawn from the market for systemic administration [117]. Site‐specific regulation of Cu ions within target tissues is critical for enhancing the therapeutic efficacy of cuproptosis and reducing off‐target effects. In this context, the development of effective Cu delivery systems is essential to establishing the applicability of cuproptosis as a novel clinical approach in cancer treatment.

Over the last two decades, a wide range of nanomaterials has been developed for biomedical purposes, such as organic carriers (e.g., polymeric nanoparticles (NPs), proteins, lipids, and micelles), inorganic systems (e.g., carbon‐based nanostructures, metal oxides, and metal–organic frameworks), and hybrid composites (e.g., core–shell and polymer–lipid hybrids) [118]. Among these, Cu‐based nanomaterials functionalized with targeting moieties represent promising candidates, as they can selectively transport Cu ions or small‐molecule inducers of cuproptosis into malignant cells while minimizing nonspecific distribution to healthy tissues [119]. These nanomaterials offer multiple advantages, including structural flexibility, excellent redox activity, biocompatibility, targeting efficiency, and responsiveness to diverse stimuli [120]. Following accumulation in the TME, these nanosystems can be disassembled by endogenous cues or external factors, thereby releasing bioactive Cu species that promote cuproptosis.

Despite their potential, the rational design of Cu‐based nanomaterials continues to face significant challenges. These include the fine‐tuning of surface ligands and controlling physicochemical parameters such as composition, morphology, particle size, and microstructural organization, as well as ensuring scalability and reproducibility in large‐scale synthesis. Although these obstacles have not been fully overcome, advancements have been made in recent years for enhanced cuproptosis‐mediated cancer therapy. This section will discuss the design and development of strategies involving various types of Cu‐based nanomaterials, including organic, inorganic, and hybrid platforms.

### 3.1 Copper‐Based Organic Nanomaterials

These nanosystems typically integrate Cu ions with organic ligands, polymers, or biomolecules, enabling precise control over particle size, surface functionality, and stability in physiological environments (Table 2).

**TABLE 2:** Overview of Cu‐based organic nanomaterials engineered to induce cuproptosis. The table summarizes the organic ligand and other biomaterials of each system, the main regulated cell death pathways activated, and the corresponding references, highlighting the relationship between NP design and biological outcome.

Nanosystem	Ligand	Biomolecule	Cell death pathway	Ref.
Cel–Cu	Celastrol	DSPE‐PEG_2000_	Cuproptosis and ICD	[121]
CuET@PHF	DSF	Polydopamine, hydroxyethyl starch, and folic acid		[122]
PCD@Cu	Camptothecin	PEG	Cuproptosis, apoptosis, and ICD	[123]
GPCuD	Tyrosine	PEG and matrix metalloproteinase‐2‐cleavable GPLGLAG peptide	Cuproptosis, apoptosis, ICD, and reprogramming the ITM	[124]
PC@B‐H	Plumbagin	HA and BSA	Cuproptosis, ferroptosis, necroptosis, and ICD	[125]
Cu–Pic	Piceatannol	HA	Cuproptosis and pyroptosis	[126]
CKPP	glutaric acid	Polydopamine, PEG, and α‐ketoglutaric acid		[127]
HA–ZCu	HA	HA	Cuproptosis and ferroptosis	[128]
5FCN	HA	chitosan and HA		[129]
MHRC@Cu	Hexahistidine	Peptide‐conjugated probe	Cuproptosis	[130]

Among the organic scaffolds employed, polyethylene glycol (PEG) is frequently used as a biomedical polymer to allow responsive drug release and improved biocompatibility [131].

Lu et al. developed self‐amplified cuproptosis NPs using the natural compound Cel. Cel can also chelate Cu through its C‐2 carbonyl and C‐3 hydroxyl groups. The authors demonstrated that the resulting Cel–Cu complex is a 1:1 complex through spectroscopic analyses. A Cel–Cu complex NP (Cel–Cu NP) was developed to overcome the limited solubility and bioavailability of the metal complex. To enhance these, the complex was encapsulated with a PEGylated phospholipid (DSPE‐PEG_2000_) to generate stable Cel–Cu NPs with a size of 110 nm [132]. These NPs exhibited superior stability in systemic circulation and preferential accumulation within tumor tissues. Once internalized, Cel–Cu NPs released both Cu and Cel. The authors evaluated Cu uptake in 4T1 breast cancer cells using CuCl_2_, the Cel + CuCl_2_ mixture, and Cel–Cu NPs for comparison. Confocal microscopy with the Cu probe rhodamine B hydrazide reveals that only cells treated with Cel–Cu NPs exhibited strong red fluorescence, indicating significant Cu accumulation. Quantitative analysis by atomic absorption spectroscopy (AAS) confirmed that the intracellular Cu content in Cel–Cu NP–treated cells was 6.4 and 3.5 times higher than in cells treated with CuCl_2_ and Cel + CuCl_2_, respectively. The released Cu ions bound to lipoylated DLAT, inducing cuproptosis, while Cel inhibited the NF‐κB pathway and depleted intracellular GSH, amplifying the cuproptotic process in a self‐reinforcing manner. Notably, Cel–Cu NP induced ICD, promoting dendritic cell maturation and the infiltration of cytotoxic T lymphocytes. When combined with immune checkpoint blockade, it effectively eliminated metastatic tumors in a mouse model of lung metastasis, demonstrating a promising nanomedicine strategy for cancer therapy.

Expanding the use of PEG‐based systems, the PCD@Cu NPs serve as an innovative therapeutic platform for TNBC by combining apoptosis, cuproptosis, and immune activation in a synergistic way [123]. Peng and colleagues synthesized two prodrugs for this purpose: PEG‐TK‐DOX, responsive to ROS, and PEG‐DTPA‐SS‐CPT, which responds to GSH. PEG‐TK‐DOX was obtained by functionalizing PEG‐diethylenetriaminepentaacetic acid (PEG‐DTPA) with camptothecin (CPT) using a disulfide bond. Then, doxorubicin (DOX) was linked to PEG with ROS‐sensitive thioketal groups (PEG‐TK‐DOX). These prodrugs self‐assembled and chelated Cu(II) ions to form PCD@Cu NPs, which carried DOX, CPT, and Cu(II) together. High levels of ROS and GSH in TNBC cells disrupted the NP structure, triggering the release of Cu(II), DOX, and CPT, together with GSH depletion. DOX and CPT caused apoptosis and ICD, while PCD@Cu downregulated ATP7B, leading to marked intracellular Cu accumulation and the onset of cuproptosis, characterized by DLAT aggregation and the loss of Fe–S cluster proteins. Transcriptomic analysis revealed that PCD@Cu reprogrammed cell metabolism by suppressing glycolysis, boosting mitochondrial respiration, and blocking cancer‐driving pathways like PI3K‐Akt, Ras, and Wnt, as well as stopping metastasis. Overall, PCD@Cu demonstrated strong antitumor effects by inducing apoptosis and cuproptosis both in vitro and in vivo, along with excellent biosafety.

In line with the previously described PEG‐based Cu nanoplatforms that synergistically integrate chemotherapy and cuproptosis, further progress has been achieved through structurally defined polymer–Cu coordination systems with improved synthetic efficiency. In this context, Haijun Yu et al. developed Cu‐based NPs (GPCuD NPs) using a streamlined synthetic strategy compared with conventional grafting approaches for polymeric polyphenols [124]. Their method relied on ring‐opening polymerization of L‐tyrosine N‐carboxyanhydrides (Tyr‐NCAs), initiated by a PEG segment bearing an MMP‐2‐cleavable peptide (GPLGLAG, G7), followed by *ortho*‐hydroxylation with 2‐iodoxybenzoic acid (IBX) to generate a polyphenol‐rich backbone capable of coordinating Cu(II) ions. This design enabled the efficient formation of Cu‐coordinated NPs with precise structural control. Loaded with doxorubicin (DOX), GP NPs released Cu(II) and DOX in response to high intracellular GSH levels and acidic pH, inducing cuproptosis and promoting macrophage repolarization from the M2 to the M1 phenotype. In vivo, GPCuD NPs suppressed 4T1 tumor growth by ∼80% and established long‐term immune memory, preventing lung metastasis [124].

The PC@B‐H nanocomplex is a GSH/pH‐responsive Cu‐based NP designed for treating oral squamous cell carcinoma (OSCC) [125]. It is synthesized in two steps: first, PLB‐Cu@BSA (PC@B) forms by coordinating plumbagin (PLB, an anticancer naphthoquinone from plants) with Cu(II) in the presence of BSA, which provides stability, solubility, and biocompatibility. The NPs (∼32 nm) exhibit increased cytotoxicity compared to PLB alone. In the second step, coating with hyaluronic acid (HA) produces PC@B‐H (∼58 nm), enabling CD44‐mediated tumor targeting. In the acidic and reducing TME, the nanocomplex releases Cu(II) and PLB, triggering cuproptosis, ferroptosis, and necroptosis. These processes elevate oxidative stress, deplete GSH, impair mitochondria, and induce ICD that enhances dendritic cell maturation and cytotoxic T cell infiltration, fostering durable antitumor immunity. In vitro, PC@B‐H shows higher activity against MOC‐1 cells compared to normal keratinocytes (HOK). The authors propose that this reduced toxicity may be due to two main factors: lower CD44 levels and GSH levels in HOK cells, which impede the internalization and degradation of the NPs, respectively. PC@B‐H demonstrates strong therapeutic potential by combining targeted drug delivery, multiple cell death pathways, and immune activation.

Another multifunctional organic nanomaterial capable of targeting CD44 through the use of HA was obtained using piceatannol (Pic) [126]. Cu–Pic NPs were synthesized via a facile one‐pot coordination assembly between Cu and the natural polyphenol Pic under mild conditions, followed by surface modification with HA to obtain Cu–Pic/HA NPs. The coordination between Cu and the phenolic hydroxyl groups of Pic led to the formation of stable Cu–O bonds, resulting in amorphous metal–phenolic nanostructures. The subsequent conjugation of HA provided a negatively charged hydrophilic shell, enhancing colloidal stability, biocompatibility, and tumor‐targeting capability. Zeta‐potential measurements showed a shift from positive to negative values after HA modification, corroborating surface encapsulation. The resulting Cu–Pic/HA NPs exhibited excellent stability in aqueous, PBS, and serum‐containing media for up to 7 days, while showing accelerated degradation under tumor‐mimicking conditions (pH 5.5, GSH, and H_2_O_2_), confirming their pH‐ and redox‐responsive biodegradability. Cu–Pic/HA NPs exhibited multiple enzyme‐mimicking activities that promote ROS generation and enhance polyamine consumption, while the released Pic inhibits Arg2‐mediated polyamine synthesis within mitochondria. Simultaneously, Cu‐induced lysosomal disruption reduced polyamine uptake by impairing the ATP13A2 transporter. The resulting total depletion of intracellular polyamines caused mitochondrial dysfunction, Cu accumulation, and protein aggregation, triggering cuproptosis. Cu–Pic/HA NPs synergistically enhance pyroptosis and also offer a promising avenue for cancer immunotherapy. Indeed, inflammasome activation and GSDMD palmitoylation lead to enhanced pyroptosis. The release of DAMPs subsequently activates the immune response, reversing tumor immunosuppression.

Among the organic systems designed for enhanced cancer immunotherapy through combined pyroptosis and cuproptosis mechanisms, CKPP NPs were engineered as a polydopamine‐coated Cu–ketoglutaric acid (KG) coordination polymer [127]. The NP works by amplifying ROS production through multiple pathways, including ketoglutaric acid‐induced mitochondrial reprogramming and polydopamine‐mediated superoxide dismutase‐like activity, while simultaneously depleting cellular GSH. Additionally, the NP shifts cancer cell metabolism from glycolysis to oxidative phosphorylation, which enhances Cu‐induced cell death. In preclinical testing using a mouse colorectal cancer model, CKPP demonstrated remarkable efficacy, achieving a 96.3% tumor inhibition rate with complete tumor eradication in two out of five cases.

In 2025, zero‐valent Cu^0^ NPs (ZCu) were designed as a multifunctional nanoplatform capable of inducing both cuproptosis and ferroptosis in tumor cells [128]. HA‐functionalized ZCu (HA‐ZCu) were synthesized via a solvothermal reaction between Cu ions and citric acid, followed by HA modification through electrostatic and coordination interactions between the carboxyl groups of HA and Cu ions. Within the acidic TME, Cu^0^ is oxidized to cuprous ions (Cu(I)), catalyzing the conversion of H_2_O_2_ into Cu ions and hydroxyl radicals (•OH), triggering cuproptosis, while the accumulation of •OH downregulates GSH peroxidase 4 (GPX4) and increases lipid peroxidation (LPO), leading to ferroptosis. This dual mechanism enhances antitumor efficacy by simultaneously activating two distinct pathways of cell death.

Another example of an organic nanosystem capable of inducing both cuproptosis and ferroptosis is 5FCN, which is based on chitosan and HA polymers [129]. The NPs are prepared using sodium tripolyphosphate as a crosslinking agent to form a chitosan/HA network, which is subsequently coordinated with Cu(II) and Fe(III) ions. The two metals are added in a 5:5 molar ratio, resulting in NPs with a hydrodynamic diameter of approximately 201 nm. 5FCN was shown to be internalized by 4T1 cells through CD44 receptor‐mediated endocytosis. Once inside the acidic intracellular environment, the NPs become protonated, leading to the release of Cu(II) and Fe(III) ions. This triggers metal accumulation, Fenton‐like reactions, and the subsequent generation of ROS, ultimately inducing both cuproptosis and ferroptosis, demonstrating in vitro cytotoxicity in 4T1 cell lines.

A recent study reported the design of CuET@PHF NPs to treat TNBC by modulating the TME [122]. This system was synthesized using a three‐step, eco‐friendly method: Cu–diethyldithiocarbamate (Cu–ET) nanocrystals were stabilized with a polydopamine (PDA) shell, further encapsulated in hydroxyethyl starch (HES) for biocompatibility and prolonged circulation, and endowed with folic acid and mercapto group to trigger targeted release. These NPs are selective and accumulate in tumor tissues, where they activate mild photothermal therapy upon laser exposure. This treatment alleviates tumor hypoxia by normalizing the tumor physical properties and boosts the effectiveness of CuET@PHF in inducing cuproptosis in cancer stem cells. The combined effects stimulate a robust antitumor immune response, helping to suppress tumor growth, prevent recurrence, and inhibit distant metastases.

Finally, MHRC@Cu NPs were developed by self‐assembling a peptide‐conjugated probe (MHRC) with Cu(II) ions [130]. The probe comprised a fluorescent unit, MeTTQ, a hexahistidine sequence for Cu(II) coordination, a polyarginine sequence for enhancing cellular membrane penetration, and a PKM2‐derived peptide that competitively inhibits the coactivator‐associated arginine methyltransferase 1 (CARM1). Upon tumor cell uptake, Cu(II) release is triggered by the destabilization of histidine–Cu(II) coordination. This occurs through protonation of histidine residues in the acidic lysosomal environment and by the local photothermal effects induced by 660 nm laser irradiation. Cu(II) is subsequently reduced to Cu(I) by FDX1. Simultaneously, MHRC‐mediated photodynamic therapy generates H_2_O_2_, which reacts with Cu(II) via a Fenton‐like mechanism to produce highly cytotoxic hydroxyl radicals (•OH). This process depletes ATP7A and intracellular GSH, facilitating sustained Cu accumulation. In parallel, MHRC competitively binds to CARM1, thereby inhibiting PKM2 methylation and reducing glycolysis while promoting mitochondrial respiration and sensitizing glycolysis‐dependent tumor cells to cuproptosis. This multifaceted strategy achieved up to 96% tumor growth inhibition, demonstrating the potential of MHRC@Cu to amplify cuproptosis and offering a promising approach for advanced tumor therapy.

Overall, Cu‐based nanosystems that combine Cu ions with organic ligands, polymers, or biomolecules show improved structural stability and controlled responsiveness in physiological conditions [132]. They rely on pH‐ and redox‐sensitive coordination mechanisms. Designing metal–ligand interactions enables multifunctional platforms in which Cu coordination geometry impacts overall performance. Polymer‐coated Cu NPs offer high drug‐loading capacity, efficient encapsulation, and controlled release, leading to effective tumor inhibition with reduced toxicity toward healthy cells. Moreover, ligand coordination and polymer coatings enhance resistance to oxidation, helping to maintain nanosystem integrity during storage and application.

### 3.2 Copper‐Based Inorganic Nanomaterials

Cu‐based inorganic nanomaterials have emerged as versatile agents for redox‐mediated cancer therapies, owing to their ability to participate in Fenton‐like reactions, interfere with intracellular metal ion homeostasis, and trigger regulated cell death pathways such as ferroptosis and cuproptosis. Different synthetic strategies have been developed to optimize their catalytic activity, tumor selectivity, and biosafety, often combining Cu with other metals, drugs, or carrier matrices. Below, representative examples are discussed.

Wang et al. [133] reported a novel nanoplatform by integrating atomically dispersed gold (Au) into degradable Cu^0^ nanocubes. The team employed a galvanic replacement method to fabricate gold–Cu nanocubes with varying atomic ratios of Au and Cu. Among them, the Au_0__.__02_Cu_0.98_ nanocubes exhibited optimal catalytic activity. X‐ray absorption near‐edge spectroscopy confirmed that both Au and Cu maintained their zero‐valent oxidation states post synthesis, indicating stable atomic dispersion. In the TME, these nanocubes facilitate the reduction of molecular oxygen (O_2_) to hydrogen peroxide (H_2_O_2_), which subsequently decomposes into hydroxyl radicals (^•^OH). This process mimics the Fenton reaction, leading to oxidative stress and tumor cell death. The Au_0__.02_Cu_0.98_ nanocubes demonstrated effective ROS generation both in vitro and in vivo, leading to significant tumor growth inhibition in orthotopic liver cancer mouse models. Additionally, these nanocubes exhibited favorable biocompatibility and were degradable, with renal clearance observed, minimizing potential long‐term toxicity. However, the therapeutic efficacy of these nanocubes remains tightly dependent on local oxygen availability, which may limit efficacy in hypoxic tumors.

Still within the framework of synergistically modulating redox balance and intracellular Cu availability, Cu‐based inorganic nanomaterials have also been investigated in the form of Cu–Zn bimetallic sulfide NPs (CZS NPs), which employ a dual redox‐targeting approach to enhance oxidative stress, as demonstrated in L929 mouse fibroblasts and 4T1 mouse breast cancer cells [134]. These ultrasmall particles (<5 nm core, 18.3  ±  2.7 nm hydrodynamic diameter) were prepared via a simple ultrasonic mixing method using Cu(II) chloride, zinc chloride, and sodium sulfide as precursors, with bovine serum albumin (BSA) for surface stabilization. Characterization confirmed the formation of CuZnS with Cu(I) and Zn(II) as the primary oxidation states, and X‐ray photoelectron spectroscopy (XPS) and energy‐dispersive X‐ray spectroscopy (EDS) analyses verified the presence of all three metals. Mechanistically, Zn(II) ions released in the acidic TME activate the NADPH oxidase pathway, depleting intracellular NADPH and limiting GSH regeneration. At the same time, lattice Cu(I) catalyzes Fenton‐like reactions, producing hydroxyl radicals (^•^OH) from H_2_O_2_ with peroxidase‐like activity. The NPs also exhibit superoxide dismutase (SOD)‐like function, generating endogenous H_2_O_2_ to maintain ROS amplification. This multienzyme‐mimicking cascade (NOX/SOD/POD) disrupts cellular redox balance and simultaneously triggers ferroptosis, cuproptosis, and apoptosis [134]. By combining NADPH/GSH depletion with enhanced ROS generation, CZS NPs represent a potent inorganic platform for metalloimmunotherapy‐assisted cancer treatment. The simplicity of the ultrasonic preparation and the small particle size are clear advantages, suggesting potential for scalable synthesis and deep tumor penetration. However, in vivo therapeutic validation remains limited, and long‐term biosafety of bimetallic sulfides has yet to be clarified.

A further evolution toward dynamic and stimulus‐responsive systems is represented by NIR‐activated nanomotors. Zhou‘s group reports the synthesis of NIR‐driven nanomotors composed of a Cu‐phyllosilicate core (CuSiO_3_) decorated with an Au–Pd shell (CuSiO_3_@Au–Pd NMs) [135]. The nanomotors were prepared by first forming Cu_2_O spheres coated with a silica shell, which was then hydrothermally converted into Cu phyllosilicate (CuSiO_3_) nanoflowers. An Au–Pd nanoalloy layer was subsequently deposited onto the surface to enhance photothermal responsiveness. Under 808‐nm laser irradiation, the particles exhibit photothermal heating and self‐thermophoretic motion, thereby enhancing their mobility. Cellular experiments (both 2‐D and 3‐D tumor spheroid MCF‐7 models) show that the nanomotors exhibit improved cellular internalization and deeper intratumour penetration under NIR actuation compared with passive controls. Importantly, the CuSiO_3_ core releases Cu(II) ions once internalized by tumor cells and initiates their reduction by intracellular GSH and Fenton‐like reactions, leading cuproptosis. The strong photothermal performance imparted by the Au–Pd nanoalloy further amplifies these oxidative and cuproptotic processes, resulting in an efficient cuproptosis‐assisted photothermal/chemodynamic (PTT–CDT) synergistic antitumor effect.

As a final example, Zhang et al. described the characteristics of CuMoO_4_ nanodots, synthesized through a straightforward hydrothermal reaction by mixing Cu and molybdate precursors under controlled temperature and pressure, producing ultrasmall, water‐dispersible particles with diameters below 10 nm that showed good stability and biodegradability in physiological media [136]. These nanodots displayed multiple enzyme‐mimicking functions, including catalase‐, peroxidase‐, and GSH peroxidase‐like activities, which enabled modulation of the redox balance by decomposing hydrogen peroxide and depleting intracellular GSH. Upon 1064 nm near‐infrared irradiation, they exhibited strong photothermal conversion efficiency (around 41%), generating local hyperthermia that synergized with ROS production. The combined catalytic and photothermal effects promoted oxidative stress, ferroptosis, and cuproptosis, resulting in potent multimodal antitumor activity, as demonstrated by in vitro assays in breast cancer cell lines MCF‐7 and 4T1, and in vivo studies in mice bearing 4T1 tumors.

Overall, these examples demonstrate how Cu‐based inorganic nanomaterials can integrate ionic release, redox catalysis, enzyme‐mimicking functions, and photothermal activation to induce cuproptosis synergistically with other forms of regulated cell death. Such multifunctional platforms offer several advantages, including high catalytic efficiency, tunable composition, structural robustness, and the possibility of spatiotemporal control through external stimuli (e.g., NIR irradiation or electrodynamic activation). Moreover, the combination of cuproptosis with ferroptosis, apoptosis, or photothermal/chemodynamic effects provides a powerful multimodal strategy that may help overcome drug resistance and enhance therapeutic efficacy. However, important challenges remain. The therapeutic outcome is often highly dependent on TME conditions (e.g., oxygen availability, pH, and redox state), which can vary significantly among tumor types and even within the same cancer tissue. Potential long‐term toxicity, incomplete biodegradability, and off‐target metal accumulation also require careful evaluation. In addition, the complexity of some nanoplatforms may hinder large‐scale reproducibility and clinical translation. Future developments should therefore focus on improving biosafety and clearance profiles, simplifying synthetic strategies, and achieving more precise control over Cu release and intracellular targeting.

### 3.3 Copper‐Based Metal–Organic Frameworks

**FIGURE 9:**
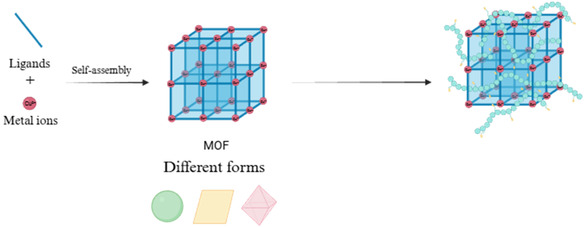
Schematic illustration of a Cu‐based metal–organic framework (MOF) composed of metal nodes coordinated with organic linkers to form a porous crystalline network of different forms. The internal cavities enable the incorporation of therapeutic agents, photosensitizers, nucleic acids, and additional metal ions, while surface functionalization allows targeted and multifunctional delivery.

Another type of platform that has been explored for the delivery of Cu is that of metal–organic frameworks (MOFs). These are crystalline porous materials composed of metal ions coordinated with complex organic linkers. MOFs are discussed separately due to their intrinsically hybrid crystalline architecture, in which metal nodes and organic linkers are integrated into a single coordination network, conferring structural and functional properties that are fundamentally distinct from those of purely inorganic or simple composite nanoplatforms. Their coordination bonds allow the formation of highly porous and crystalline structures with tunable morphologies and functionalities. MOFs can be synthesized under mild conditions, enabling the incorporation of diverse functional moieties (Figure 9).

**FIGURE 10:**
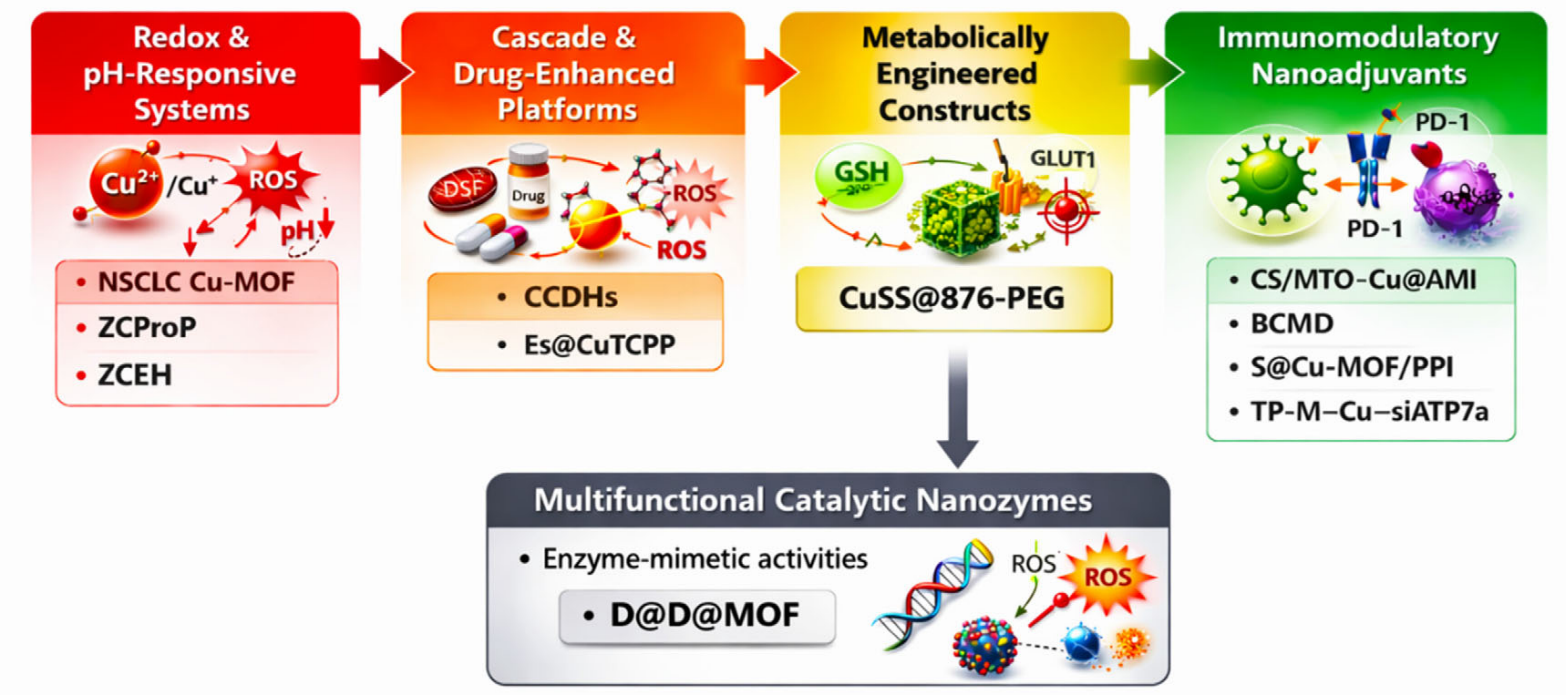
Schematic classification of Cu‐MOF systems based on their primary therapeutic design strategy. All platforms share the ability to release bioactive Cu ions and induce cuproptosis, while differing in structural design and functional approach.

The advantages of MOFs for biomedical applications include high porosity, large surface area, and, in some cases, weak coordination bonds that render them biodegradable, as well as their ability to achieve controlled and sustained drug delivery, maintaining therapeutic levels over extended periods. Biocatalytic and biosensing capabilities as metal centers can act as catalytic sites for enzymatic and nonenzymatic reactions. High selectivity and functional versatility allowing targeted detection of specific cells or biomolecules. Structural tunability, stability, and lipophilicity permitting optimization for physiological environments or TME responsiveness, facilitating interaction with biological membranes, and enhancing the delivery of hydrophobic drugs [137, 138, 139]. Numerous systems have been developed in an effort to exploit the advantageous features of MOFs in the biomedical field. Although all Cu‐MOF systems share the ability to release bioactive Cu ions and induce cuproptosis, they can be broadly categorized according to their primary therapeutic design strategy (Figure 10). A first group includes pH‐ or redox‐responsive Cu‐MOFs that rely mainly on intracellular Cu release and Fenton‐like reactions to trigger oxidative damage, apoptosis, and cuproptosis. A second category encompasses drug‐loaded or cascade‐amplified systems, in which Cu release is combined with chemotherapeutics, ionophores, or nanozyme‐like activities to enhance ROS production and mitochondrial dysfunction. More advanced designs exploit tumor metabolic vulnerabilities, integrating Cu‐mediated cuproptosis with mechanisms such as disulfidptosis through targeted inhibition of glucose uptake or GSH metabolism. Finally, immunomodulatory Cu‐MOF platforms couple cuproptosis induction with immune activation, including cGAS–STING pathway stimulation or PD‐L1 modulation, thereby transforming Cu overload into a trigger for systemic antitumor immunity. This functional classification highlights the progressive evolution of Cu‐MOFs from simple Cu‐delivery systems to multifunctional platforms capable of orchestrating complex therapeutic cascades. It should be noted that this classification is functionally oriented, primarily conceptual, and intended to enhance clarity, rather than to impose rigid boundaries, as several Cu‐based MOF systems integrate overlapping mechanisms, such as redox amplification, metabolic interference, and immune activation, reflecting the intrinsically multifunctional nature of these nanoplatforms. We summarize below representative examples of Cu‐based MOF systems, discussing at least one illustrative case for each functional category.

A study applied a lysosome‐responsive Cu‐MOF as a cuproptosis‐mediated nanoplatform for the treatment of NSCLC, particularly KRAS‐mutated tumors [140]. Upon endocytic uptake, the MOF disassembles within the acidic lysosomal microenvironment, enabling controlled Cu ion release. The liberated Cu(II) ions catalyze Fenton‐type reactions, generating hydroxyl radicals that induce oxidative damage, cytoskeletal disruption, and caspase‐3 activation, thereby triggering apoptosis. In parallel, Cu accumulation within mitochondria, mediated by FDX1, promotes binding to DLAT, leading to ISC protein loss and aggregation of lipoylated proteins, ultimately resulting in proteotoxic stress‐driven cuproptosis. The simultaneous activation of apoptosis and cuproptosis produced significant antitumor effects both in vitro and in vivo, highlighting how pH‐responsive Cu‐MOFs can effectively engage multiple regulated cell death pathways.

A targeted approach was proposed by Deng et al. [141], who engineered ZCProP, a MOF system designed for mitochondrial delivery of Cu and prodigiosin (Pro), a cytotoxic bright red tripyrrole pigment from *Serratia marcescens*. The ZCProP NPs were obtained using zeolitic imidazolate framework‐90 (ZIF‐90) as the core, encapsulating Cu(II), Pro, and PEG modification within a pH/ATP‐responsive matrix. The ZCProP NPs exhibited a diameter of approximately 60 nm and a zeta potential of around −15 mV. In vitro studies on 4T1 cells demonstrated that ZCProP NPs accumulated in the mitochondria of the tumor cells. Upon exposure to acidic pH and elevated ATP levels, conditions prevalent in the TME, they underwent structural destabilization, leading to the controlled release of Cu(II) and Pro [141]. The liberated Cu(II) promoted aggregation of lipoylated mitochondrial proteins and depletion of ISC proteins, leading to activation of cuproptosis. Concurrently, intracellular GSH depletion further amplified oxidative stress, facilitating both cuproptosis and ferroptosis through suppression of GSH peroxidase 4 (GPX4). In parallel, Pro contributed to cytotoxicity by inducing cellular stress and intercalating into double‐stranded DNA. In the presence of Cu, Pro can form a 1:1 coordination complex in which the three pyrrolic nitrogen atoms interact with the metal center. Cu‐mediated oxidative transformation of the C‐pyrrole ring, including hydroxylation at C1 and rearrangement of conjugation within the tripyrrolic scaffold, has been reported [142]. The resulting Cu–Pro complex is believed to enhance oxidative DNA damage, thereby further contributing to tumor cell death [143, 144].

Targeted antibody‐functionalized MOFs also contribute to the landscape of pH‐ or redox‐responsive Cu‐MOFs. ZCEH, based on a Zr–Cu MOF loaded with ES and conjugated with trastuzumab, represents another pH‐sensitive system designed to selectively induce cuproptosis in HER2‐positive tumors, combining Cu‐mediated mitochondrial dysfunction with receptor‐directed targeting to enhance therapeutic precision [145]. ZCEH is based on a Zr‐Cu MOF that was hydrothermally synthesized by coassembling ZrCl_4_, terephthalic acid, and polyvinylpyrrolidone (PVP) in DMF, followed by the addition of CuCl_2_ and heating. The resulting hexagonal prism NPs (∼120 nm hydrodynamic diameter) exhibited hierarchical porosity with a high surface area. Zr‐Cu MOF was loaded with ES and coated with trastuzumab through carbodiimide chemistry, utilizing the surface carboxyl groups. Also in this case, the results suggest selectivity, biosafety, and effective therapeutic action both in vitro and in vivo.

Moving beyond purely Cu‐release–driven platforms, the second category encompasses drug‐loaded and cascade‐amplified Cu‐MOF systems, in which coordinated therapeutics or catalytic components are integrated to potentiate oxidative stress and enhance cuproptosis efficacy.

Exploiting controlled Cu release, CCDHs are composed of a Cu_2_O core coated with a CuBTC MOF shell, a Cu‐based MOF constructed with benzene‐1,3,5‐tricarboxylate linkers [146]. The shell is generated through a sacrificial growth process, where the MOF develops by leaching from the Cu_2_O surface. This strategy allows for a surface and MOF layer that are more reactive and less stable, promoting a more efficient release of Cu(I) ions. DSF was loaded by exploiting the porosity of the MOF shell, whereas a coating with HA endowed the system with hydrophilicity and tumor‐targeting capability mediated by the CD44 receptor. The CuBTC MOF shell stabilizes the Cu_2_O core while enabling rapid dissolution in acidic environments (pH ∼ 5.5), but the system remains stable at neutral pH, thereby ensuring stability during circulation. In vitro, CCDHs exhibit low toxicity toward normal cells but induce cuproptosis in tumor cells, with DSF enhancing ROS production, mitochondrial damage, and overall therapeutic efficacy. In vivo studies confirm tumor‐targeted accumulation, high biosafety, and potent antitumor effects, achieving up to 93.1% tumor growth inhibition in subcutaneous tumor models.

Another example of a tumor‐responsive MOF is the nanosheet Es@CuTCPP, based on porphyrins and ES [147]. These nanostructures were synthesized via a solvothermal reaction in which Cu(II) ions were coordinated with tetrakis(4‐carboxyphenyl)porphyrin (TCPP), followed by the incorporation of ES [148]. The drug was loaded through noncovalent interactions, primarily hydrophobic forces and π–π interactions within the porphyrin matrix, resulting in uniform nanosheets with an average thickness of ∼5 nm and lateral dimensions of 200–300 nm. The Es@CuTCPP nanostructures were evaluated in the murine colon adenocarcinoma cell line CT26, including 3D mammospheres enriched for cancer stem‐like cells (CSCs). Elevated GSH levels in tumor cells trigger the redox conversion of Cu(II) to Cu(I), making the nanosheets sonosensitive; upon ultrasound, they generate ROS, while codelivered ES forms a Cu–ES complex that induces cuproptosis in CSCs. In vivo studies using a CT26 colon cancer model showed that Es@CuTCPP rapidly accumulated at tumor sites and remained there for at least 24 h, demonstrating efficient tumor targeting with minimal off‐target accumulation. Moreover, long‐term monitoring in healthy mice revealed stable vital parameters and no organ damage, indicating a low systemic toxicity of the nanomedicine nanoplatform. Preclinical data suggest that Es@CuTCPP offers tumor‐selective, dual‐action therapy through ROS generation and cuproptosis, though its efficacy may depend on tumor GSH levels, and the need for ultrasound may limit clinical translation.

Another nanoplatform called BCMD was developed based on MOF‐199, a Cu(II)‐based MOF composed of paddlewheel clusters interconnected by benzene‐1,3,5‐tricarboxylate linkers, forming a highly porous three‐dimensional network [149]. In this architecture, Cu is an integral structural component, while the system is further loaded with buthionine‐sulfoximine (BSO) and catalase (CAT) through weak interactions. BSO and CAT can act to decrease GSH and increase O_2_, respectively. Moreover, dodecyl‐β‐D‐maltoside, an absorption promoter, was coated on the surface of the NPs. The final NPs exhibited a hydrodynamic diameter (DLS data) of approximately 115 nm, whereas the size was ∼50 nm (TEM analysis) for unfunctionalized MOF‐199. BMDC demonstrated pH‐responsive release behavior, with rapid cargo liberation under acidic conditions (pH 5.5) characteristic of the TME. The bioactivity of BCMD was first tested in vitro. In GL261 murine glioma cells, BCMD caused a pronounced loss of ISP proteins, as demonstrated through Western blot analysis, along with the formation of DLAT foci, indicating DLAT aggregation. These findings confirmed that cell death was mediated by cuproptosis. Notably, a selective behavior of BCMD was evident, as mouse brain endothelial cells (bEnd.3) displayed negligible cytotoxicity under the same conditions. To rule out contributions from other cell death pathways, a panel of inhibitors targeting ferroptosis (ferrostatin‐1), necroptosis (necrostatin‐1), oxidative stress (N‐acetylcysteine), and apoptosis (Z‐VAD‐FMK) was tested, but none prevented Cu‐induced death, confirming cuproptosis as the main mechanism. In vivo studies were performed in a murine GBM model using GL261‐luc cells inoculated intracranially. BCMD was administered intranasally, facilitated by dodecyl‐β‐D‐maltoside as an absorption promoter. ICP analysis confirmed significant Cu accumulation in tumor tissue after treatment. Therapeutic efficacy was evidenced by tumor suppression and prolonged survival. At the same time, histological and immunological analyses indicated that cuproptosis mediated by BCMD further promoted ICD, stimulating dendritic cell activation and enhancing T cell infiltration into the TME. Notably, biosafety analysis demonstrated excellent tolerability, with no detectable systemic toxicity. Overall, these findings establish BCMD as a safe and effective nanoplatform capable of selectively inducing cuproptosis in glioma cells while simultaneously boosting antitumor immunity.

Another representative example is the TP‐M–Cu–MOF/siATP7a system, designed for targeted therapy of small‐cell lung cancer [150]. It consists of a Cu‐based MOF, incorporating a pH‐responsive imidazole‐containing ligand (4,4′‐BIDBA) and cloaking the NPs with a mesenchymal stem cell (MSC) membrane functionalized with the TP0751 peptide, derived from *Treponema pallidum* (the causative agent of syphilis). The platform achieves enhanced tumor targeting and improved permeability across biological barriers, including the BBB. Finally, the system also transports siRNA against ATP7a to modulate Cu homeostasis and inhibit tumor invasiveness. The stem cell membrane coating significantly enhanced NP uptake by tumor cells, as demonstrated by higher fluorescence intensity in M–Cu–MOF/siRNA and TP‐M–Cu–MOF/siRNA compared with uncoated Cu–MOF/siRNA at the same concentration. Quantitative flow cytometry analysis in human small‐cell lung cancer cell line H69 confirmed that membrane modification promotes preferential internalization by cancer cells. To evaluate the NP ability to cross barriers, an in vitro model (using bEnd.3 endothelial cells) was employed. TP0751‐decorated NPs showed higher transport across the endothelial layer than unmodified M–Cu–MOF/siRNA, indicating that surface functionalization substantially augments NP permeability. Once internalized, TP‐M–Cu–MOF/siATP7A reduces FDX1 levels, suggesting the induction of cuproptosis. The siRNA‐mediated silencing of ATP7A could reduce tumor invasiveness and epithelial–mesenchymal transition. TP‐M–Cu–MOF/siATP7A effectively kills H69 cells while sparing normal cells, thanks to high tumor GSH levels and MSC membrane–mediated targeting. In vivo investigations in an SCLC brain metastasis mouse model were also encouraging. The TP‐M–Cu–MOF/siATP7A NPs were administered intravenously every 3 days for a total of 6 doses. In addition to being effective in inhibiting tumor growth and reducing tumor size, TP‐M–Cu–MOF/siATP7A did not influence blood biochemical parameters that remained within normal ranges, indicating minimal liver or kidney toxicity [150].

Beyond single‐mechanism designs, more complex immunomodulatory platforms have been developed and based on CD44 targeting. One example is CS/MTO–Cu@AMI, designed to improve chemo‐immunotherapy by overcoming two significant cancer mechanisms of resistance: macropinocytosis and intracellular bacteria [151]. The nanoplatform combines mitoxantrone (MTO, a cytotoxic, chemotherapeutic, and immunogenic agent) coordinated with Cu(II) ions to form an MOF (MTO–Cu). This structure is then loaded with amiloride (AMI), an inhibitor of macropinocytosis and exosome secretion, and with chondroitin sulfate (CS) for CD44 targeting. CS/MTO–Cu@AMI showed strong antitumor effects against drug‐resistant MCF‐7 breast cancer cells, demonstrating promising potential for overcoming chemotherapy resistance. In contrast, normal immortalized human liver cells (LO2) and human kidney epithelial cells (293T) maintained high viability even at the highest drug concentration, indicating low toxicity and good biocompatibility of the system for cancer therapy. Mechanistically, the coordinated Cu(II) ions trigger cuproptosis, leading to mitochondrial dysfunction, oxidative stress, and AMPK‐mediated degradation of PD‐L1. At the same time, AMI inhibits macropinocytosis and exosome release, working together with Cu(II) to enhance therapeutic effectiveness. The treatment also causes double‐stranded DNA damage, activates the cGAS–STING pathway, and enhances systemic antitumor immunity. The platform is pH‐ and GSH‐responsive, releasing more MTO and AMI in the presence of GSH and at pH 5.0, with hyaluronidase‐triggered charge reversal ensuring selective tumor accumulation and minimal toxicity to healthy tissues. In experiments, CS/MTO–Cu@AMI was tested in 4T1 breast cancer cells at specific time points to evaluate cytotoxicity, cellular uptake, and immune activation, and further tested in vivo in 4T1 tumor‐bearing mice. Given the high metastatic potential of 4T1 cells, the authors also examined the antimetastatic effect in the lungs. A mouse model of lung metastasis was created through tail vein injection of luc‐4T1 cells, and bioluminescence imaging showed that treatment with CS/MTO–Cu@AMI effectively suppressed lung metastasis. Histological analysis confirmed that the fewest metastatic lesions were present in the CS/MTO–Cu@AMI group, supporting the platform's capacity to generate immune memory responses capable of preventing recurrence and metastasis after surgery. Overall, this rationally designed nanoadjuvant effectively sensitizes chemotherapy, activates systemic antitumor immunity, and suppresses metastasis, showing great promise as a tumor‐selective immune amplifier capable of eradicating both primary and metastatic cancers with minimal side effects.

Morphology‐driven enhancement strategies further expand this field. Xu et al. developed a spiky S@Cu‐MOF functionalized with polyphyllin I, yielding NPs with a rambutan‐like morphology and an average size of ∼186 nm [152]. Its rambutan‐like surface (Spiky morphology) enhances cellular uptake through endocytosis. This design couples Cu‐induced mitochondrial dysfunction and DLAT aggregation with DNA damage and activation of the cGAS/STING pathway, synergizing with αPD‐1 immunotherapy and significantly improving antitumor immune responses.

Although CRUPPA19 is based on a Zr‐MOF rather than a Cu‐MOF, its therapeutic mechanism aligns with immunomodulatory Cu‐MOF platforms, as Cu‐mediated cuproptosis is functionally integrated with immune activation [153]. In this system, a Cu–Rhein complex is encapsulated within the amine‐functionalized zirconium framework UiO‐66‐NH_2_, forming a nanoscale carrier further modified with mPEG‐PO_3_ and anti‐CD19 antibodies to enable selective targeting of B‐cell lymphoma (BCL) cells, including those infiltrating the bone marrow. Confocal microscopy and flow cytometry confirmed preferential internalization in CD19‐positive A20 lymphoma cells, with negligible uptake in normal monocytes, highlighting its targeting specificity. Upon exposure to acidic tumor microenvironment conditions and ultrasound stimulation, CRUPPA19 undergoes autophagy‐mediated degradation, leading to controlled release of Cu ions and Rhein. The liberated Cu enhances mitochondrial protein lipoylation and promotes DLAT aggregation, driving FDX1‐dependent cuproptosis, while Rhein induces global mRNA hypermethylation and transcriptional repression of PD‐L1. These coordinated events stimulate ICD, activate CD8^+^ T cells, and generate a robust systemic antitumor immune response. In murine lymphoma models, CRUPPA19‐mediated sono‐immunotherapy effectively eradicated both primary and metastatic lesions, including bone marrow‐resident tumor cells, demonstrating favorable biodistribution, tumor accumulation, and significant therapeutic efficacy.

Unlike previous Cu‐MOF systems primarily focused on redox amplification or immunomodulation, CuSS@876‐PEG rationally exploits tumor metabolic reprogramming to induce dual cuproptosis–disulfidptosis synthetic lethality. Chemically, the platform consists of a Cu‐based MOF engineered with disulfide‐containing components that enable GSH‐responsive behavior. CuSS@876‐PEG is constructed from Cu(II) ions coordinated with dithiodiglycolic acid as the organic linker. The ligand incorporates a disulfide (–S–S–) unit directly within the framework backbone, generating a redox‐responsive coordination network. XPS spectra indicate the coexistence of Cu(II) and Cu(I) species, and TEM images show a uniform polygonal nanoflower morphology with an average diameter of ∼86 nm. Surface functionalization with DSPE‐mPEG reduced aggregation, yielding CuSS@876‐PEG NPs with a hydrodynamic diameter of ∼141 nm and improved colloidal stability. The framework remains structurally stable under mildly acidic conditions but undergoes disassembly under reductive environments due to cleavage of the disulfide linkages, resulting in release of Cu species and encapsulated BAY‐876. Upon intracellular uptake, the elevated GSH levels typical of SLC7A11‐overexpressing tumors promote partial framework degradation, leading to GSH consumption and controlled release of Cu(II) ions. This redox‐triggered Cu liberation facilitates cuproptosis. Simultaneously, the nanoplatform delivers BAY‐876, a selective GLUT1 inhibitor, which suppresses glucose uptake and induces metabolic stress. In the context of high SLC7A11 expression and glucose dependency, this glucose deprivation promotes disulfidptosis, a form of cell death associated with cytoskeletal disulfide stress. The PEG modification enhances colloidal stability and prolongs systemic circulation, improving tumor accumulation. Biochemically, the combined depletion of GSH, induction of Cu overload, and inhibition of glucose metabolism amplifies oxidative stress and triggers ICD, thereby coupling metabolic vulnerability with immune activation [154].

An emerging and particularly innovative category is that of MOF‐based nanozymes. These are synthetic nanomaterials that replicate enzymatic functions while overcoming the limitations of natural enzymes, such as poor stability, high cost, and limited scalability. Their tunable catalytic activities, robustness, and ability to modulate ROS within TME make them promising candidates for next‐generation cancer therapies. The dextran‐coated Cu‐MOF nanozyme termed D@D@MOF, coordinated with 3‐amino‐1,2,4‐triazole and loaded with DSF, integrates enzyme‐mimetic activities (peroxidase‐, SOD‐, and GPx‐like) with catalase inhibition [155]; the researchers showed that the cytotoxicity of D@D@MOF is mainly mediated by cuproptosis. Inhibition with the Cu chelator TTM significantly reduced cell death, while inhibitors of apoptosis and ferroptosis had little impact. In vitro, using the 4T1 cell line, the treatment induced significant ROS accumulation, GSH depletion, mitochondrial dysfunction, including loss of membrane potential, along with downregulation of cuproptosis‐related proteins such as DLAT and LIAS. In contrast, when tested on normal cells such as HUVECs, the NP did not compromise cell viability. In vivo, in TNBC tumor‐bearing animal models, D@D@MOF achieved marked tumor growth inhibition with minimal systemic toxicity. These findings suggest that the nanozyme exerts negligible cytotoxicity toward healthy cells, thereby supporting its favorable biosafety profile.

In summary, Cu‐based MOF platforms have emerged as a mature and increasingly sophisticated class of therapeutic systems. Several designs already show tumor selectivity through intrinsic microenvironment responsiveness, surface modifications, or biomimicry. Their modular architecture allows for controlled Cu release, multimodal therapy, and synergistic activation of cuproptosis along with other mechanisms. The main challenge is therefore shifting from proof‐of‐concept to practical, clinical application: this involves long‐term biosafety studies, precise control of systemic Cu redistribution, scalable and reproducible manufacturing processes, and standardized in vivo testing. Future efforts will likely focus on enhancing pharmacokinetic predictability, simplifying designs without losing functionality, and ensuring material engineering meets regulatory standards. With ongoing optimization and thorough validation, Cu‐MOF systems hold real potential for clinical use, beyond just experimental nanoplatforms.

### 3.4 Copper‐Based Hybrid Nanomaterials

Cu‐based hybrid nanomaterials have recently attracted increasing attention in oncology because they integrate the intrinsic redox and cuproptosis‐inducing properties of Cu with the complementary structural and functional features of organic or inorganic components, thereby overcoming several limitations of single‐component systems.

A first structural class comprises inorganic scaffold‐based hybrids, in which mesostructured or layered hosts physically confine catalytic or ionophoric components. The dendritic mesoporous silica system (DLMSN) developed by Wan et al. exemplifies this design by developing a multifunctional platform combining Cu release and electrodynamic therapy. DLMSN was synthesized via a dual‐templating method [156]. Tetraethyl orthosilicate (TEOS) served as the silica precursor, yielding spherical NPs with large, radially oriented mesopores. Pt(IV) ions (PtCl_6_^2‐^) were then adsorbed electrostatically into the DLMSN pores and reduced with NaBH_4_ to form Pt NPs within the pores, yielding the “nano‐pomegranate” (N‐PG) particles. N‐PG particles were then loaded with NSC by adsorption; the drug loading content was approximately 10%, and the encapsulation efficiency was about 50%. Microneedles were then fabricated by photopolymerization of methacrylate‐based HA with N‐PG/NSC, yielding a crosslinked polymer matrix with high mechanical integrity, ensuring that the particles are stably stored until the microneedles penetrate the oral mucosa. Compared to conventional injections or topical application, this strategy offers several advantages, including protecting the NPs from premature aggregation or degradation, ensuring a controlled and localized release upon tissue insertion, minimizing systemic dispersion, and enhancing patient compliance for noninvasive, pain‐free therapy. To improve colloidal stability in a physiological environment, N‐PG/NSC particles were dispersed in PVP, which substantially reduced aggregation and sedimentation in PBS over 24 h. Under application conditions, the N‐PG particles exhibit good electrochemical stability. After 20 min of electrolysis using the square‐wave alternating current employed in electrodynamic therapy (EDT, 0.1 Hz, 8 mA), their visible light absorption remained almost unchanged, demonstrating that they can sustain ROS generation through EDT without substantial degradation. To study cellular internalization, the authors replaced NSC with fluorescein isothiocyanate (FITC) to generate N‐PG/FITC, then exposed SCC‐7 oral carcinoma cells to free FITC or N‐PG/FITC [156]. Flow cytometry and confocal microscopy showed time‐dependent uptake: more particles were internalized over 2–4 h, N‐PG/FITC significantly more than free FITC. This platform was tested in vitro on the cell line SCC‐7 and in vivo on the murine OSCC. During EDT, Pt NPs promote water dissociation, generating highly reactive ^•^OH that attack key biomolecules, including nucleic acids, proteins, and membrane lipids, and cause structural damage to organelles such as mitochondria, the endoplasmic reticulum, and lysosomes, ultimately triggering cell death. In parallel, the Cu ionophore NSC319726 enhances intracellular Cu(II) uptake, induces DLAT aggregation, and depletes iron–sulfur cluster proteins. These events drive cuproptosis.

Layered double hydroxide (LDH)‐based systems represent a second inorganic hybrid configuration in which Cu is embedded within a lamellar host lattice that enables both ion exchange and stimulus‐responsive release [157]. Hydrothermally synthesized CuAl‐LDH nanosheets exhibit a well‐defined hexagonal sheet‐like morphology with lateral dimensions of 100–200 nm and a thickness of 4.5–5.0 nm. Structural and compositional homogeneity (uniform distribution of Cu, Al, and O) was confirmed by TEM, AFM, XRD, and XPS analyses. The positively charged brucite‐like layers and interlayer galleries allow the incorporation of 5‐fluorouracil (5‐FU) via ion‐exchange processes, while subsequent adsorption of HA on the surface affords LDH/HA/5‐FU nanosheets with an average hydrodynamic diameter of 324 nm, low polydispersity (PDI 0.13), and stable zeta potential, indicating good colloidal stability. Under acidic tumor‐mimicking conditions, partial protonation and lattice destabilization of the LDH framework promote the coordinated release of Cu(II) and 5‐FU. The liberated Cu ions participate in Fenton‐like reactions, leading to GSH depletion and hydroxyl radical (^•^OH) generation, while the simultaneously released chemotherapeutic agent enhances cytotoxic stress. This synchronized release profile couples chemodynamic therapy (CDT) with chemotherapy (CT) within a single lamellar inorganic scaffold. Moreover, HA functionalization confers CD44‐mediated cellular uptake, contributing to selective internalization and enabling modulation of the tumor microenvironment, as reflected by increased M1‐like tumor‐associated macrophages and enhanced CD4^+^/CD8^+^ T‐cell infiltration alongside reduced M2 polarization.

A related but structurally distinct inorganic hybrid strategy is exemplified by ZnO_2_@Cu NPs, which employ a core–shell redox cascade architecture. In this system, a ZnO_2_ core is encapsulated within a Cu‐ion‐doped polydopamine shell. In acidic tumor microenvironments, the ZnO_2_ core gradually decomposes, releasing H_2_O_2_ as a sustained endogenous ROS precursor. The elevated intratumoral H_2_O_2_ concentration in turn accelerates degradation of the polydopamine shell, facilitating exposure and release of Cu ions. These Cu species react with H_2_O_2_ to trigger sequential redox cycling reactions that amplify oxidative stress and culminate in cuproptosis, while also exhibiting synergy with radiotherapy [158]. In both LDH‐ and ZnO_2_‐based systems, the hierarchical inorganic architecture governs not only Cu availability but also the temporal sequence of redox events. Lamellar ion‐exchange lattices and degradable core–shell structures thus provide chemically programmed pathways for synchronized ion release, ROS amplification, and spatially confined Cu‐mediated reactivity.

A distinct category includes polymer‐coordinated Cu hybrids, where organic frameworks define Cu coordination geometry and release behavior. In 2025, porous organic polymer (POP) NPs were engineered to deliver and selectively accumulate Cu(II) within tumor cells to potentiate cuproptosis. The POPs, incorporating bipyridine moieties, were synthesized on an aminated silica template, followed by coordination with Cu(II) and loading of the sonosensitizer artesunate (ART), yielding Cu/ART@Hpy NPs [159]. The resulting NPs had a hydrodynamic size of roughly 150 nm and a zeta potential of +30 mV, with an ART loading efficiency of approximately 15%. In the acidic TME, these NPs exhibited pH‐responsive release of Cu(II), while ultrasound irradiation triggered the generation of ROS, depleting intracellular GSH and enhancing Cu(II) accumulation. This dual mechanism not only induced cuproptosis but also activated multiple cell death pathways, highlighting the potential of Cu/ART@Hpy NPs as a platform for sonodynamically enhanced tumor therapy.

Finally, lipid–metal nanocomposites provide a biomimetic hybrid architecture. In CuP/Er NPs, a Cu‐ and peroxide‐containing core is encapsulated within a lipid bilayer [160]. CuP/Er comprises a core loaded with Cu ions and peroxide, surrounded by a shell of 1,2‐dioleoyl‐sn‐glycero‐3‐phosphocholine (DOPC), cholesterol, and DSPE‐PEG2K. Incorporating erastin (Er) in an equimolar ratio to Cu during the coating process results in the formation of the bifunctional NP. In 4T1 cells, CuP/Er induced a disruption of lysosomal integrity and mitochondrial Cu accumulation, sensitizing tumor cells to cuproptosis by interfering with aerobic glycolysis and impairing the TCA cycle via oligomerization of DLAT. At the same time, the NP promotes ferroptosis by increasing ROS levels and disrupting intracellular redox homeostasis, leading to GSH depletion, elevated lipid peroxidation, and severe mitochondrial damage. These combined effects translated into potent tumor growth inhibition in murine breast and colon cancer models. In addition, CuP/Er was found to induce ICD, enhancing antigen presentation and upregulating PD‐L1 expression on tumor cells. When combined with anti‐PD‐L1 therapy, CuP/Er produced potent antitumor effects, not only regressing primary tumor size but also limiting metastatic spread. This configuration demonstrates how soft‐matter encapsulation can modulate Cu speciation and integrate multiple redox‐active species within a single nanoscale construct.

From a chemical perspective, these hybrid systems share a unifying principle: Cu reactivity is governed not solely by ion release, but by the structural context in which Cu is embedded. Mesoporous confinement, layered ion‐exchange lattices, polymeric chelation, and lipid encapsulation each impose distinct coordination environments and degradation pathways, thereby controlling Cu redox cycling, ROS generation, and downstream biochemical reactivity. In this sense, Cu‐based hybrid nanomaterials exemplify how rational materials design can transform a simple transition‐metal ion into a stimulus‐responsive catalytic module within multifunctional nanostructures.

## 4 Limitations and Drawbacks

The studies discussed above establish cuproptosis as a mechanistically distinct and increasingly tractable strategy for cancer therapy [161]. However, its clinical translation remains constrained by fundamental challenges associated with the control of Cu biology and nanomaterial behavior [162]. A central limitation lies in the insufficient regulation of Cu homeostasis and speciation, which directly impacts both therapeutic efficacy and safety. The redox‐active nature of Cu, while essential for triggering cell death, imposes a narrow therapeutic window due to the risk of off‐target oxidative damage. This is further exacerbated by the dynamic and competitive biological environment, where endogenous ligands can sequester Cu, thereby compromising delivery efficiency and predictability. The interactions with copper‐binding biomolecules, e.g., amino acids, GSH, metallothioneins, and serum proteins such as HSA, should be thoroughly investigated to validate these systems and more accurately predict their in vivo behavior in terms of stability, biodistribution, and copper release.

In parallel, the selectivity of cuproptosis‐inducing systems remains suboptimal. Many agents rely on tumor‐associated triggers, including elevated GSH, ROS, or acidic pH, that are inherently heterogeneous across tumor types and patients, leading to variable activation and inconsistent therapeutic responses. This lack of precision is compounded by the propensity of Cu to accumulate in nontarget tissues, which may induce mitochondrial dysfunction, disrupt oxidative metabolism, and activate alternative cell‐death pathways. Moreover, systemic Cu dysregulation can interfere with essential cellular processes, including ISC stability and metal homeostasis networks, amplifying toxicity beyond the intended therapeutic scope.

Additional concerns arise from the immunological consequences of Cu‐based therapies. While localized Cu‐induced stress may enhance ICD, uncontrolled or systemic exposure can trigger off‐target inflammatory responses and immune dysregulation, posing further safety risks. The development of advanced delivery strategies, such as NP‐based carriers or antibody–drug conjugates, could be promising platforms for minimizing systemic toxicity and improving tumor targeting [163].

From a nanomedicine perspective, these challenges are compounded by limitations common to nanoscale systems, including suboptimal biodistribution, accumulation within the reticuloendothelial system, and difficulties in achieving scalable and reproducible synthesis of complex multicomponent architectures. The stability of Cu–ligand coordination under physiological conditions remains difficult to maintain, and premature or uncontrolled metal release can undermine therapeutic precision. Furthermore, repeated administration may elicit immunogenic responses, particularly in PEGylated formulations, complicating long‐term treatment strategies.

Collectively, these limitations highlight that, despite their considerable promise, cuproptosis‐based systems require more precise control over Cu speciation, improved targeting strategies, and a deeper understanding of their systemic biological interactions to enable safe and effective clinical translation.

## 5 Conclusions

This review examined the potential of cuproptosis as an emerging paradigm in cancer therapy, focusing on its unique biochemical basis and its significance as a novel therapeutic target. It dedicated particular attention to the rational design of systems that induce cuproptosis, emphasizing how coordination chemistry, redox reactivity, ligand engineering, and materials architecture collectively influence Cu speciation and intracellular activity. By adopting a predominantly chemical and biochemical perspective, often underrepresented in current literature, this work aimed to contextualize recent advances beyond just therapeutic applications. The review thus offers a conceptual framework for developing more refined systems capable of safely and effectively harnessing cuproptosis for anticancer applications. A detailed and critically structured overview of Cu ionophores explored in anticancer approaches has been provided, ranging from classical, well‐established Cu‐binding agents (ES and DSF) to more recently developed compounds, including Cuphoralix, illustrating the evolution in structural design and mechanistic understanding in this area. The therapeutic potential of these compounds is particularly relevant in the context of aggressive cancers like TNBC, where conventional treatments often fall short due to drug resistance and high heterogeneity. The application of cuproptosis‐related gene signatures in aggressive cancers highlights the potential for personalized treatment approaches that align therapies with individual tumor profiles, offering hope for improved patient outcomes.

NP‐based delivery systems have emerged as a critical advancement in overcoming the limitations of traditional cuproptosis inducers. NPs enable targeted delivery and sustained release of Cu ions within the TME, enhancing the accumulation of Cu in cancer cells and improving therapeutic outcomes. The synergy between cuproptosis inducers and immune checkpoint inhibitors, such as αPD‐L1, further highlights the potential for combination therapies to enhance anti‐tumor activity and address resistance mechanisms. The ability of NPs to overcome physiological barriers and to improve drug delivery represents a significant step forward in cancer therapy, offering new opportunities for effective treatment.

Despite these advancements, several challenges remain in the clinical application of cuproptosis‐based therapies. One primary concern is the lack of targeting specificity of Cu‐based agents, which can lead to toxicity in normal tissues and limit the effectiveness of treatment. Additionally, the induction of cuproptosis can cause mitochondrial dysfunction, impacting cells with high energy demands and potentially exacerbating patient conditions. Addressing the challenges of cuproptosis‐based therapies requires ongoing research. The development of more selective Cu‐based agents and advanced delivery systems that minimize off‐target effects and maximize therapeutic efficacy is crucial. The refinement of NP technologies and targeted therapies will be essential for overcoming the current limitations and improving the clinical utility of cuproptosis‐based treatments. Continued research and development are essential to harnessing the full potential of cuproptosis to improve patient survival and quality of life.

## Funding

This study was supported by ERMES (grant 101185661), and Università degli Studi di Catania.

## Conflicts of Interest

The authors declare no conflicts of interest.

## Data Availability

Data sharing not applicable to this article as no datasets were generated or analysed during the current study.

## References

[1] Y. Fu , L. Hou , K. Han , et al., “The Physiological Role of Copper: Dietary Sources, Metabolic Regulation, and Safety Concerns,” Clinical Nutrition (edinburgh, Scotland) 48 (2025): 161–179, 10.1016/j.clnu.2025.03.023. 40220473

[2] D. Tang , G. Kroemer , and R. Kang , “Targeting Cuproplasia and Cuproptosis in Cancer,” Nature Reviews. Clinical Oncology 21 (2024): 370–388, 10.1038/s41571-024-00876-0. 38486054

[3] V. C. Shanbhag , N. Gudekar , K. Jasmer , et al., “Copper Metabolism as a Unique Vulnerability in Cancer,” Biochimica Et Biophysica Acta. Molecular Cell Research 1868 (2021): 118893, 10.1016/j.bbamcr.2020.118893. 33091507 PMC7779655

[4] E. J. Ge , A. I. Bush , A. Casini , et al., “Connecting Copper and Cancer: From Transition Metal Signalling to Metalloplasia,” Nature Reviews Cancer 22 (2022): 102–113, 10.1038/s41568-021-00417-2. 34764459 PMC8810673

[5] P. Tsvetkov , S. Coy , B. Petrova , et al., “Copper Induces Cell Death by Targeting Lipoylated TCA Cycle Proteins,” Science (new York, N.y.) 375 (2022): 1254–1261, 10.1126/science.abf0529. 35298263 PMC9273333

[6] Y. Wang , Y. Chen , J. Zhang , et al., “Cuproptosis: A Novel Therapeutic Target for Overcoming Cancer Drug Resistance,” Drug Resistance Updates: Reviews and Commentaries in Antimicrobial and Anticancer Chemotherapy 72 (2024): 101018, 10.1016/j.drup.2023.101018. 37979442

[7] L. Chen , J. Min , and F. Wang , “Copper Homeostasis and Cuproptosis in Health and Disease,” Signal Transduction and Targeted Therapy 7 (2022): 378, 10.1038/s41392-022-01229-y. 36414625 PMC9681860

[8] Y. Feng , Z. Yang , J. Wang , and H. Zhao , “Cuproptosis: Unveiling a New Frontier in Cancer Biology and Therapeutics,” Cell Communication and Signaling: Ccs 22 (2024): 249, 10.1186/s12964-024-01625-7. 38693584 PMC11064406

[9] S. Sun , J. Cai , Q. Yang , et al., “The Association between Copper Transporters and the Prognosis of Cancer Patients Undergoing Chemotherapy: A Meta‐Analysis of Literatures and Datasets,” Oncotarget 8 (2016): 16036–16051, 10.18632/oncotarget.13917. PMC5362544 27980217

[10] M.‐H. Khadem‐Ansari , M. Asoudeh , H. F. K. Gheshlaghi , et al., “Copper and Zinc in Stage I Multiple Myeloma: Relation with Ceruloplasmin, Lipid Peroxidation, and Superoxide Dismutase Activity, Hormone Molecular Biology and Clinical Investigation 37 (2019): 20180055, j/hmbci.2019.37.issue‐3/hmbci‐2018‐0055/hmbci‐2018‐0055.xml, 10.1515/hmbci-2018-0055. 30367794

[11] A. Gupte and R. J. Mumper , “Elevated Copper and Oxidative Stress in Cancer Cells as a Target for Cancer Treatment,” Cancer Treatment Reviews 35 (2009): 32–46, 10.1016/j.ctrv.2008.07.004. 18774652

[12] D. M. Medeiros , and D. Jennings , “Role of Copper in Mitochondrial Biogenesis Via Interaction with ATP Synthase and Cytochrome c Oxidase,” Journal of Bioenergetics and Biomembranes 34 (2022): 389–395, 10.1023/A:1021206220851. 12539966

[13] S. Lutsenko , S. Roy , and P. Tsvetkov , “Mammalian Copper Homeostasis: Physiological Roles and Molecular Mechanisms,” Physiological Reviews 105 (2025): 441–491, 10.1152/physrev.00011.2024. 39172219 PMC11918410

[14] Y. Fu , S. Zeng , Z. Wang , et al., “Mechanisms of Copper‐Induced Autophagy and Links with Human Diseases,” Pharmaceuticals 18 (2025): 99, 10.3390/ph18010099. 39861161 PMC11768742

[15] H. Li , Y. An , X. Luo , et al., “Cuproptosis Accompanied by Obvious •OH Generation Revealed with an Ultrasensitive NIR Fluorescence Probe,” Chemical Engineering Journal (lausanne, Switzerland: 1996) 476 (2023): 146749, 10.1016/j.cej.2023.146749.

[16] O. Y. Dmitriev and J. Patry , “Structure and Mechanism of the Human Copper Transporting ATPases: Fitting the Pieces into a Moving Puzzle,” Biochimica et Biophysica Acta (BBA) ‐ Biomembranes 1866 (2024): 184306, 10.1016/j.bbamem.2024.184306. 38408697

[17] X. Li , Z. Dai , J. Liu , et al., “Characterization of the Functional Effects of ferredoxin 1 as a Cuproptosis Biomarker in Cancer,” Frontiers in Genetics 13 (2022): 969856, 10.3389/fgene.2022.969856. 36226187 PMC9549589

[18] M. B. Dreishpoon , N. R. Bick , B. Petrova , et al., “FDX1 Regulates Cellular Protein Lipoylation through Direct Binding to LIAS,” Journal of Biological Chemistry 299 (2023): 105046, 10.1016/j.jbc.2023.105046. 37453661 PMC10462841

[19] A. Trivedi , J. Bakhasha , V. Saxena , et al., “Understanding Cuproptosis through FDX1‐Mediated Lipoylation and Proteotoxic Stress in *Channa Punctatus* ,” Journal of Hazardous Materials Advances 19 (2025): 100788, 10.1016/j.hazadv.2025.100788.

[20] J. Lin , G. Wang , S. Cheng , et al., “Pan‐Cancer Analysis of the Cuproptosis‐Related Gene DLD,” Mediators of Inflammation 2023 (2023): 5533444, 10.1155/2023/5533444. 38077227 PMC10703539

[21] Q. Lou , F. Lai , J. Li , K. Mao , H. Wan , and Y. He , “Mechanisms of Cuproptosis and its Relevance to Distinct Diseases,” Apoptosis 29 (2024): 981–1006, 10.1007/s10495-024-01983-0. 38824478

[22] C. Springer , D. Humayun , and R. Skouta , “Cuproptosis: Unraveling the Mechanisms of Copper‐Induced Cell Death and Its Implication in Cancer Therapy,” Cancers 16 (2024): 647, 10.3390/cancers16030647. 38339398 PMC10854864

[23] X. Tong , R. Tang , M. Xiao , et al., “Targeting Cell Death Pathways for Cancer Therapy: Recent Developments in Necroptosis, Pyroptosis, Ferroptosis, and Cuproptosis Research,” Journal of Hematology & Oncology 15 (2022): 174, 10.1186/s13045-022-01392-3. 36482419 PMC9733270

[24] K. M. Abdullah , J. B. Kaushal , S. Takkar , et al., “Copper Metabolism and Cuproptosis in Human malignancies: Unraveling the Complex Interplay for Therapeutic Insights,” Heliyon 10 (2024): e27496, 10.1016/j.heliyon.2024.e27496. 38486750 PMC10938126

[25] S. J. Dixon and J. A. Olzmann , “The Cell Biology of Ferroptosis,” Nature Reviews Molecular Cell Biology 25 (2024): 424–442, 10.1038/s41580-024-00703-5. 38366038 PMC12187608

[26] C. Berndt , H. Alborzinia , V. S. Amen , et al., “Ferroptosis in Health and Disease,” Redox Biology 75 (2024): 103211, 10.1016/j.redox.2024.103211. 38908072 PMC11253697

[27] X. Jin , J. Tang , X. Qiu , et al., “Ferroptosis: Emerging Mechanisms, Biological Function, and Therapeutic Potential in Cancer and Inflammation,” Cell Death Discovery 10 (2024): 45, 10.1038/s41420-024-01825-7. 38267442 PMC10808233

[28] J. Wang , J. Li , J. Liu , et al., “Interplay of Ferroptosis and Cuproptosis in Cancer: Dissecting Metal‐Driven Mechanisms for Therapeutic Potentials,” Cancers 16 (2024): 512, 10.3390/cancers16030512. 38339263 PMC10854932

[29] N. Liu and M. Chen , “Crosstalk between Ferroptosis and Cuproptosis: From Mechanism to Potential Clinical Application,” Biomedicine & Pharmacotherapy 171 (2024): 116115, 10.1016/j.biopha.2023.116115. 38181713

[30] Y. Gou , M. Chen , S. Li , et al., “Dithiocarbazate‐Copper Complexes for Bioimaging and Treatment of Pancreatic Cancer,” Journal of Medicinal Chemistry 64 (2021): 5485–5499, 10.1021/acs.jmedchem.0c01936. 33861929

[31] K. Li , K. Xu , Y. He , et al., “Oxygen Self‐Generating Nanoreactor Mediated Ferroptosis Activation and Immunotherapy in Triple‐Negative Breast Cancer,” ACS Nano 17 (2023): 4667–4687, 10.1021/acsnano.2c10893. 36861638

[32] M. Chu , X. An , C. Fu , et al., “Disulfiram/Copper Induce Ferroptosis in Triple‐Negative Breast Cancer Cell Line MDA‐MB‐231,” Frontiers in Bioscience‐Landmark 28 (2023): 186, 10.31083/j.fbl2808186. 37664913

[33] Y. Li , F. Chen , J. Chen , et al., “Disulfiram/Copper Induces Antitumor Activity against Both Nasopharyngeal Cancer Cells and Cancer‐Associated Fibroblasts through ROS/MAPK and Ferroptosis Pathways,” Cancers 12 (2020): 138, 10.3390/cancers12010138. 31935835 PMC7017005

[34] N. Sharma , S. Dey , S. Singh , et al., “Disulfiram‐Copper Potentiates Anticancer Efficacy of Standard Chemotherapy Drugs in Bladder Cancer Animal Model through ROS‐Autophagy‐Ferroptosis Signalling Cascade, Current Cancer Drug Targets 25 (2025): 1145–1157, 10.2174/0115680096325879240815105227. 39323342

[35] C. Arkan and D. Akcora‐Yildiz , “FDA‐Approved Disulfiram Induces Ferroptosis via Alteration of Redox Balance and Lipid Peroxidation and Overcomes Carfilzomib Resistance in Multiple Myeloma,” Leukemia & Lymphoma 66 (2025): 250–261, 10.1080/10428194.2024.2422843. 39527722

[36] D.‐H. Cai , B.‐F. Liang , B.‐H. Chen , et al., “A Novel Water‐Soluble Cu(II) Gluconate Complex Inhibits Cancer Cell Growth by Triggering Apoptosis and Ferroptosis Related Mechanisms,” Journal of Inorganic Biochemistry 246 (2023): 112299, 10.1016/j.jinorgbio.2023.112299. 37354603

[37] W.‐F. Song , J.‐Y. Zeng , P. Ji , et al., “Self‐Assembled Copper‐Based Nanoparticles for Glutathione Activated and Enzymatic Cascade‐Enhanced Ferroptosis and Immunotherapy in Cancer Treatment,” Small (weinheim an Der Bergstrasse, Germany) 19 (2023): 2301148, 10.1002/smll.202301148. 37118853

[38] W. Wang , K. Lu , X. Jiang , et al., “Ferroptosis Inducers Enhanced Cuproptosis Induced by Copper Ionophores in Primary Liver Cancer,” Journal of Experimental & Clinical Cancer Research 42 (2023): 142, 10.1186/s13046-023-02720-2. 37277863 PMC10242978

[39] Q. Xue , R. Kang , D. J. Klionsky , et al., “Copper Metabolism in Cell Death and Autophagy,” Autophagy 19 (2023): 2175–2195, 10.1080/15548627.2023.2200554. 37055935 PMC10351475

[40] Y. Zhang , Q. Jia , J. Li , et al., “Copper‐Bacteriochlorin Nanosheet as a Specific Pyroptosis Inducer for Robust Tumor Immunotherapy,” Advanced Materials (deerfield Beach, Fla.) 35 (2023): 2305073, 10.1002/adma.202305073. 37421648

[41] V. Oliveri , “Biomedical Applications of Copper Ionophores,” Coordination Chemistry Reviews 422 (2020): 213474, 10.1016/j.ccr.2020.213474.

[42] V. Oliveri , “Selective Targeting of Cancer Cells by Copper Ionophores: An Overview,” Frontiers in Molecular Biosciences 9 (2022), 10.3389/fmolb.2022.841814. PMC8931543 35309510

[43] N. H. Vo , Z. Xia , J. Hanko , et al., “Synthesis, Crystallographic Characterization and Electrochemical Property of a Copper(II) Complex of the Anticancer Agent Elesclomol,” Journal of Inorganic Biochemistry 130 (2014): 69–73. 10.1016/j.jinorgbio.2013.10.005. 24176921

[44] M. Zulkifli , A. N. Spelbring , Y. Zhang , et al., “FDX1‐Dependent and Independent Mechanisms of Elesclomol‐Mediated Intracellular Copper Delivery,” Proceedings of the National Academy of Sciences of the United States of America 120 (2023): e2216722120, 10.1073/pnas.2216722120. PMC10013847 36848556

[45] P. Zheng , C. Zhou , L. Lu , et al., “Elesclomol: A Copper Ionophore Targeting Mitochondrial Metabolism for Cancer Therapy,” Journal of Experimental & Clinical Cancer Research 41 (2022): 271, 10.1186/s13046-022-02485-0. 36089608 PMC9465867

[46] J. Gao , X. Wu , S. Huang , et al., “Novel Insights into Anticancer Mechanisms of Elesclomol: More than a Prooxidant Drug,” Redox Biology 67 (2023): 102891, 10.1016/j.redox.2023.102891. 37734229 PMC10518591

[47] A. A. Yadav , D. Patel , X. Wu , and B. B. Hasinoff , “Molecular Mechanisms of the Biological Activity of the Anticancer Drug Elesclomol and its Complexes with Cu(II), Ni(II) and Pt(II),” Journal of Inorganic Biochemistry 126 (2013): 1–6, 10.1016/j.jinorgbio.2013.04.013. 23707906

[48] N. M. Garza , M. Zulkifli , and V. M. Gohil , “Elesclomol Elevates Cellular and Mitochondrial Iron Levels by Delivering Copper to the Iron Import Machinery,” Journal of Biological Chemistry 298 (2022): 102139, 10.1016/j.jbc.2022.102139. 35714767 PMC9270252

[49] L. W. Njenga , S. N. Mbugua , R. A. Odhiambo , and M. O. Onani , “Addressing the Gaps in Homeostatic Mechanisms of Copper and Copper Dithiocarbamate Complexes in Cancer Therapy: A Shift From Classical Platinum‐Drug Mechanisms,” Dalton Transactions (cambridge, England: 2003) 52 (2023): 5823–5847, 10.1039/D3DT00366C. 37021641

[50] F. P. Andrew and P. A. Ajibade , “Metal Complexes of Alkyl‐Aryl Dithiocarbamates: Structural Studies, Anticancer Potentials and Applications as Precursors for Semiconductor Nanocrystals,” Journal of Molecular Structure 1155 (2018): 843–855, 10.1016/j.molstruc.2017.10.106.

[51] G. Hogarth and D. C. Onwudiwe , “Copper Dithiocarbamates: Coordination Chemistry and Applications in Materials Science, Biosciences and Beyond,” Inorganics 9 (2021): 70, 10.3390/inorganics9090070.

[52] J. Lanz , N. Biniaz‐Harris , M. Kuvaldina , et al., “Disulfiram: Mechanisms, Applications, and Challenges,” Antibiotics 12 (2023): 524, 10.3390/antibiotics12030524. 36978391 PMC10044060

[53] H. Li , J. Wang , C. Wu , et al., “The Combination of Disulfiram and Copper for Cancer Treatment. Drug Discov Today,” Drug Discovery Today 25 (2020): 1099–1108, 10.1016/j.drudis.2020.04.003. 32320854

[54] S. Zhang , Y. Zong , L. Chen , et al., “The Immunomodulatory Function and Antitumor Effect of Disulfiram: Paving the Way for Novel Cancer Therapeutics,” Discover Oncology 14 (2023): 103, 10.1007/s12672-023-00729-9. 37326784 PMC10275851

[55] V. Kannappan , M. Ali , B. Small , et al., “Recent Advances in Repurposing Disulfiram and Disulfiram Derivatives as Copper‐Dependent Anticancer Agents,” Frontiers in Molecular Biosciences 8 (2021), 10.3389/fmolb.2021.741316. PMC8484884 34604310

[56] M. Zeng , B. Wu , W. Wei , et al., “Disulfiram: A Novel Repurposed Drug for Cancer Therapy, Chinese Medical Journal 137 (2024): 1389–1398, 10.1097/CM9.0000000000002909. 38275022 PMC11188872

[57] Q. Yang , Y. Yao , K. Li , et al., “An Updated Review of Disulfiram: Molecular Targets and Strategies for Cancer Treatment, Current Pharmaceutical Design 25 (2019): 3248–3256, 10.2174/1381612825666190816233755. 31419930

[58] Y. Li , L.‐H. Wang , H.‐T. Zhang , et al., “Disulfiram Combined with Copper Inhibits Metastasis and Epithelial–mesenchymal Transition in Hepatocellular Carcinoma through the NF‐κB and TGF‐β Pathways,” Journal of Cellular and Molecular Medicine 22 (2018): 439–451, 10.1111/jcmm.13334. 29148232 PMC5742719

[59] X. Ren , Y. Li , Y. Zhou , et al., “Overcoming the Compensatory Elevation of NRF2 Renders Hepatocellular Carcinoma Cells More Vulnerable to Disulfiram/Copper‐Induced Ferroptosis,” Redox Biology 46 (2021): 102122, 10.1016/j.redox.2021.102122. 34482117 PMC8416961

[60] D. Nie , C. Chen , Y. Li , and C. Zeng , “Disulfiram, an Aldehyde Dehydrogenase Inhibitor, Works as a Potent Drug against Sepsis and Cancer via NETosis, Pyroptosis, Apoptosis, Ferroptosis, and Cuproptosis,” Blood Science (baltimore, Md.) 4 (2022): 152–154, 10.1097/BS9.0000000000000117. 36518588 PMC9742096

[61] Y. Gan , T. Liu , W. Feng , et al., “Drug Repositioning of Disulfiram Induces Endometrioid Epithelial Ovarian Cancer Cell Death via the Both Apoptosis and Cuproptosis Pathways,” Oncology Research 31 (2023): 333–343, 10.32604/or.2023.028694. 37305383 PMC10229305

[62] P. Zhang , C. Zhou , X. Ren , et al., “Inhibiting the Compensatory Elevation of xCT Collaborates with Disulfiram/Copper‐Induced GSH Consumption for Cascade Ferroptosis and Cuproptosis,” Redox Biology 69 (2024): 103007, 10.1016/j.redox.2023.103007. 38150993 PMC10788306

[63] N. Huang , Y. Feng , Y. Liu , et al., “Disulfiram Mediated Anti‐Tumour Effect in Pituitary Neuroendocrine Tumours by Inducing Cuproptosis,” International Immunopharmacology 134 (2024): 112159, 10.1016/j.intimp.2024.112159. 38692018

[64] P. Li , Q. Sun , S. Bai , et al., “Combination of the Cuproptosis Inducer Disulfiram and Anti‐PD‐L1 Abolishes NSCLC Resistance by ATP7B to Regulate the HIF‐1 Signaling Pathway,” International Journal of Molecular Medicine 53 (2024): 1–9, 10.3892/ijmm.2023.5343. 38186308 PMC10781418

[65] T. S. Lobana , R. Sharma , G. Bawa , and S. Khanna , “Bonding and Structure Trends of Thiosemicarbazone Derivatives of Metals—An Overview,” Coordination Chemistry Reviews 253 (2009): 977–1055, 10.1016/j.ccr.2008.07.004.

[66] M. B. Paterson , and S. P. Donnelly , “Copper Complexes of Bis(thiosemicarbazones): From Chemotherapeutics to Diagnostic and Therapeutic Radiopharmaceuticals,” Chemical Society Reviews 40 (2011): 3005–3018, 10.1039/C0CS00215A. 21409228

[67] X.‐G. Bai , Y. Zheng , and J. Qi , “Advances in Thiosemicarbazone Metal Complexes as Anti‐Lung Cancer Agents,” Frontiers in Pharmacology 13 (2022), 10.3389/fphar.2022.1018951. PMC9551402 36238553

[68] C. Stefani , Z. Al‐Eisawi , P. J. Jansson , et al., “Identification of Differential Anti‐Neoplastic Activity of Copper Bis(thiosemicarbazones) that Is Mediated by Intracellular Reactive Oxygen Species Generation and Lysosomal Membrane Permeabilization,” Journal of Inorganic Biochemistry 152 (2015): 20–37, 10.1016/j.jinorgbio.2015.08.010. 26335599

[69] A. Li , K. Huang , W. Pan , et al., “Thiosemicarbazone Mixed‐Valence Cu(I/II) Complex against Lung Adenocarcinoma Cells through Multiple Pathways Involving Cuproptosis,” Journal of Medicinal Chemistry 67 (2024): 9091–9103, 10.1021/acs.jmedchem.4c00257. 38778566

[70] M. Dharmasivam , M. G. Azad , R. Afroz , et al., “The Thiosemicarbazone, DpC, Broadly Synergizes with Multiple Anti‐Cancer Therapeutics and Demonstrates Temperature‐ and Energy‐Dependent Uptake by Tumor Cells,” Biochimica et Biophysica Acta (BBA) ‐ General Subjects 1866 (2022): 130152, 10.1016/j.bbagen.2022.130152. 35436509

[71] A. E. Stacy , D. Palanimuthu , P. V. Bernhardt , et al., “Zinc(II)–Thiosemicarbazone Complexes are Localized to the Lysosomal Compartment Where They Transmetallate with Copper Ions to Induce Cytotoxicity,” Journal of Medicinal Chemistry 59 (2016): 4965–4984, 10.1021/acs.jmedchem.6b00238. 27023111

[72] S. Paukovcekova , J. Skoda , J. Neradil , et al., “Novel Thiosemicarbazones Sensitize Pediatric Solid Tumor Cell‐Types to Conventional Chemotherapeutics through Multiple Molecular Mechanisms,” Cancers 12 (2020): 3781, 10.3390/cancers12123781. 33334021 PMC7765366

[73] Northwestern University , A Phase 1 Adaptive Dose Escalation With Dose Expansion Study of Triapine in Combination With Temozolomide (TMZ) for Patients With Recurrent Glioblastoma, 2025, https://clinicaltrials.gov.

[74] National Cancer Institute (NCI) , A Phase I Trial Combining Triapine With Radiation Therapy for Recurrent Glioblastoma or Astrocytoma, 2025, https://clinicaltrials.gov.

[75] National Cancer Institute , Phase I Dose‐Escalation Bioavailability Study of Oral Triapine in Combination With Concurrent Chemoradiation for Locally Advanced Cervical Cancer (LACC) and Vaginal Cancer, 2025, https://clinicaltrials.gov.

[76] National Cancer Institute , A Randomized Phase III Trial of Radiation Therapy and Cisplatin Alone or in Combination With Intravenous Triapine in Women With Newly Diagnosed Bulky Stage IB2, Stage II, IIIB, or IVA Cancer of the Uterine Cervix or Stage II‐IVA Vaginal Cancer, 2025, https://Clinicaltrials.gov.

[77] K. Ishiguro , Z. P. Lin , P. G. Penketh , et al., “Distinct Mechanisms of Cell‐Kill by Triapine and Its Terminally Dimethylated Derivative Dp44mT due to a Loss or Gain of Activity of Their Copper(II) Complexes,” Biochemical Pharmacology 91 (2014): 312–322, 10.1016/j.bcp.2014.08.006. 25130544 PMC4163625

[78] K. C. Park , L. Fouani , P. J. Jansson , et al., “Copper and Conquer: Copper Complexes of Di‐2‐Pyridylketone Thiosemicarbazones as Novel Anti‐Cancer Therapeutics,” Metallomics: Integrated Biometal Science 8 (2016): 874–886, 10.1039/c6mt00105j. 27334916

[79] A. E. Stacy , D. Palanimuthu , P. V. Bernhardt , et al., “Structure–Activity Relationships of Di‐2‐Pyridylketone, 2‐Benzoylpyridine, and 2‐Acetylpyridine Thiosemicarbazones for Overcoming Pgp‐Mediated Drug Resistance,” Journal of Medicinal Chemistry 59 (2016): 8601–8620, 10.1021/acs.jmedchem.6b01050. 27524608

[80] M. Dharmasivam , B. Kaya , P. Wijesinghe , T , et al., “Differential Transmetallation of Complexes of the Anti‐Cancer Thiosemicarbazone, Dp4e4mT: Effects on Anti‐Proliferative Efficacy, Redox Activity, Oxy‐Myoglobin and Oxy‐Hemoglobin Oxidation,” Chemical Science 15 (2024): 974–990, 10.1039/D3SC05723B. 38239703 PMC10793205

[81] J. Hu , Y. Guo , R. Mao , et al., “Copper Complexes with Quinoline‐Thiosemicarbazone Hybrid Ligand Promoting Apoptosis by Causing DNA and Mitochondria Dual Lesions,” Journal of Molecular Structure 1314 (2024): 138719, 10.1016/j.molstruc.2024.138719.

[82] K. Shimada , E. Reznik , M. E. Stokes , et al., “Copper‐Binding Small Molecule Induces Oxidative Stress and Cell Cycle Arrest in Glioblastoma‐Patient‐Derived Cells,” Cell Chemical Biology 25 (2018): 585–594.e7, 10.1016/j.chembiol.2018.02.010. 29576531 PMC5959763

[83] K. L. Summers , M. J. Pushie , G. J. Sopasis , et al., “Solution Chemistry of Copper(II) Binding to Substituted 8‐Hydroxyquinolines, Inorganic Chemistry 59 (2020): 13858–13874, 10.1021/acs.inorgchem.0c01356. 32936627

[84] V. Oliveri , V. Lanza , D. Milardi , et al., “Amino‐ and Chloro‐8‐Hydroxyquinolines and Their Copper Complexes as Proteasome Inhibitors and Antiproliferative Agents,” Metallomics: Integrated Biometal Science 9 (2017): 1439–1446, 10.1039/c7mt00156h. 28932850

[85] S. H. Liang , A. G. Southon , B. H. Fraser , et al., “Novel Fluorinated 8‐Hydroxyquinoline Based Metal Ionophores for Exploring the Metal Hypothesis of Alzheimer's Disease,” ACS Medicinal Chemistry Letters 6 (2015): 1025–1029, 10.1021/acsmedchemlett.5b00281. 26396692 PMC4569883

[86] W.‐Q. Ding and S. E. Lind , “Metal Ionophores – An Emerging Class of Anticancer Drugs,” IUBMB Life 61 (2009): 1013–1018, 10.1002/iub.253. 19859983

[87] S. Tardito , A. Barilli , I. Bassanetti , et al., “Copper‐Dependent Cytotoxicity of 8‐Hydroxyquinoline Derivatives Correlates with Their Hydrophobicity and Does Not Require Caspase Activation,” Journal of Medicinal Chemistry 55 (2012): 10448–10459, 10.1021/jm301053a. 23170953

[88] A. Steinbrueck , A. C. Sedgwick , J. T. Brewster , et al., “Transition Metal Chelators, pro‐Chelators, and Ionophores as Small Molecule Cancer Chemotherapeutic Agents,” Chemical Society Reviews 49 (2020): 3726–3747, 10.1039/C9CS00373H. 32525153

[89] W.‐Q. Ding , B. Liu , J. L. Vaught , et al., “Anticancer Activity of the Antibiotic Clioquinol,” Cancer Research 65 (2005): 3389–3395, 10.1158/0008-5472.CAN-04-3577. 15833873

[90] M. E. Helsel and K. J. Franz , “Pharmacological Activity of Metal Binding Agents that Alter Copper Bioavailability,” Dalton Transactions (cambridge, England: 2003) 44 (2015): 8760–8770, 10.1039/C5DT00634A. 25797044 PMC4425619

[91] V. Oliveri and G. Vecchio , “8‐Hydroxyquinolines in Medicinal Chemistry: A Structural Perspective,” European Journal of Medicinal Chemistry 120 (2016): 252–274, 10.1016/j.ejmech.2016.05.007. 27191619

[92] V. Oliveri , “New Glycoconjugates for the Treatment of Diseases Related to Metal Dyshomeostasis,” ChemistryOpen 4 (2015): 792–795, 10.1002/open.201500155. 27308206 PMC4906504

[93] A. Sambugaro , R. Po , M. Lorenzetto , et al., “Exploring the Effect of Copper on the Bioactivity of 8‐Quinolines: An In Vitro and In Vivo Study,” Dalton Transactions (cambridge, England: 2003) 54 (2025): 14396–14406, 10.1039/D5DT01396H. 40926669

[94] G. Pastuch‐Gawołek and J. Szreder , “Effect of Glycoconjugation on Cytotoxicity and Selectivity of 8‐Aminoquinoline Derivatives Compared to 8‐Hydroxyquinoline,” Molecules (basel, Switzerland) 30 (2025): 427, 10.3390/molecules30020427. 39860296 PMC11767929

[95] C. Moison , D. Gracias , J. Schmitt , et al., “SF3B1 Mutations Provide Genetic Vulnerability to Copper Ionophores in Human Acute Myeloid Leukemia,” Science Advances 10 (2024): eadl4018, 10.1126/sciadv.adl4018. 38517966 PMC10959413

[96] J. Jiang , G. Chen , W. Zhang , et al., “Pseudonatural Flavonols as Novel Copper Ionophores for NAFLD Intervention via Synergistic Copper Delivery and Flavonoid Activity,” Journal of Medicinal Chemistry 68 (2025): 6450–6461, 10.1021/acs.jmedchem.4c02927. 40080431

[97] Y. Ji , F. Dai , and B. Zhou , “Designing Salicylaldehyde Isonicotinoyl Hydrazones as Cu(II) Ionophores with Tunable Chelation and Release of Copper for Hitting Redox Achilles Heel of Cancer Cells,” Free Radical Biology & Medicine 129 (2018): 215–226, 10.1016/j.freeradbiomed.2018.09.017. 30240704

[98] L. Yu , Q. Shen , X. Xie , et al., “Rational Design of Copper Ionophores for Efficient Induction of Cuproptosis via Simple n‐Alkyl Modification,” European Journal of Medicinal Chemistry 301 (2026): 118257, 10.1016/j.ejmech.2025.118257. 41075604

[99] F. Dai , W.‐J. Yan , Y.‐T. Du , et al., “Structural Basis, Chemical Driving Forces and Biological Implications of Flavones as Cu(II) Ionophores,” Free Radical Biology & Medicine 108 (2017): 554–563, 10.1016/j.freeradbiomed.2017.04.023. 28431962

[100] X.‐Z. Bao , F. Dai , X.‐R. Li , and B. Zhou , “Targeting Redox Vulnerability of Cancer Cells by Prooxidative Intervention of a Glutathione‐Activated Cu(II) pro‐Ionophore: Hitting Three Birds with One Stone,” Free Radical Biology & Medicine 124 (2018): 342–352, 10.1016/j.freeradbiomed.2018.06.021. 29935260

[101] V. P. Papageorgiou , A. N. Assimopoulou , E. A. Couladouros , et al., “The Chemistry and Biology of Alkannin, Shikonin, and Related Naphthazarin Natural Products,” Angewandte Chemie International Edition 38 (1999): 270–301, 10.1002/(SICI)1521-3773(19990201)38:33.0.CO;2-0 29711637

[102] X.‐Z. Bao , Q. Wang , X.‐R. Ren , et al., “A Hydrogen Peroxide‐Activated Cu(II) pro‐Ionophore Strategy for Modifying Naphthazarin as a Promising Anticancer Agent with High Selectivity for Generating ROS in HepG2 Cells over in L02 Cells,” Free Radical Biology & Medicine 152 (2020): 597–608, 10.1016/j.freeradbiomed.2019.12.001. 31805398

[103] Y.‐L. Zheng , Y. Ji , Y. Li , et al., “Identification of Tanshinone I as a Natural Cu(II) Ionophore,” Free Radical Biology & Medicine 227 (2025): 27–41, 10.1016/j.freeradbiomed.2024.11.049. 39613045

[104] A. Mittal , M. Nagpal , V. K. Vashistha , et al., “Recent Advances in the Antioxidant Activity of Metal‐Curcumin Complexes: A Combined Computational and Experimental Review,” Free Radical Research 58 (2024): 11–26, 10.1080/10715762.2023.2298857. 38145454

[105] A. Arenaza‐Corona , M. A. Obregón‐Mendoza , W. Meza‐Morales , et al., “The Homoleptic Curcumin–Copper Single Crystal (ML2): A Long Awaited Breakthrough in the Field of Curcumin Metal Complexes,” Molecules (basel, Switzerland) 28 (2023): 6033, 10.3390/molecules28166033. 37630284 PMC10458717

[106] Y. Yang , S. Liang , H. Geng , et al., “Proteomics Revealed the Crosstalk between Copper Stress and Cuproptosis, and Explored the Feasibility of Curcumin as Anticancer Copper Ionophore,” Free Radical Biology & Medicine 193 (2022): 638–647, 10.1016/j.freeradbiomed.2022.11.023. 36395954

[107] L. Feng , T.‐Z. Wu , X.‐R. Guo , et al., “Discovery of Natural Resorcylic Acid Lactones as Novel Potent Copper Ionophores Covalently Targeting PRDX1 to Induce Cuproptosis for Triple‐Negative Breast Cancer Therapy,” ACS Central Science 11 (2025): 357–370, 10.1021/acscentsci.4c02188. 40028362 PMC11869127

[108] R. Xue , C. Qin , L. Li , et al., “SRF/SLC31A1 Signaling Promotes Cuproptosis Induced by Celastrol in NSCLC,” International Immunopharmacology 148 (2025): 114165, 10.1016/j.intimp.2025.114165. 39930648

[109] Y. Fu , L. Hou , K. Han , et al., “Epigallocatechin Gallate Promotes Cuproptosis via the MTF1/ATP7B Axis in Hepatocellular Carcinoma,” Cells 14 (2025): 391, 10.3390/cells14060391. 40136640 PMC11941326

[110] D. Li , H. Wang , L. Dai , et al., “Copper(II) Complexes of Pyrazino [2,3‐F][1,10]Phenanthroline Induce Cuprotosis and Immunogenic Cell Death in Triple‐Negative Breast Cancer,” Applied Organometallic Chemistry 39 (2025): e70324, 10.1002/aoc.70324.

[111] Y.‐Y. Ling , Q.‐H. Shen , L. Hao , et al., “Theranostic Rhenium Complexes as Suborganelle‐Targeted Copper Ionophores To Stimulate Cuproptosis for Cancer Immunotherapy,” ACS Applied Materials & Interfaces 17 (2025): 15237–15249, 10.1021/acsami.5c01443. 40025808

[112] N. Renier , G. Weyckmans Mele , P. Lelièvre , et al., “Potent Biological Activity by a Synthetic Cu(I) Cationophore Redistributing Intracellular Copper Pools,” Journal of the American Chemical Society 148 (2026): 593–604, 10.1021/jacs.5c15335. 41431421

[113] P. Lelievre , C. Nogier , N. Renier , et al., “Abstract 4681: Unlocking the Anticancer Potential of Calix[4]arene‐Based Cu(I) Ionophores In Vitro and In Vivo,” Cancer Research 84 (2024): 4681, 10.1158/1538-7445.AM2024-4681.

[114] S. Xu , Y. Hao , X. Xu , et al., “Antitumor Activity and Mechanistic Insights of a Mitochondria‐Targeting Cu(I) Complex,” Journal of Medicinal Chemistry 67 (2024): 7911–7920, 10.1021/acs.jmedchem.3c02018. 38709774

[115] M. V. Babak , D. Ahn , “Modulation of Intracellular Copper Levels as the Mechanism of Action of Anticancer Copper Complexes: Clinical Relevance,” Biomedicines 9 (2021): 852, 10.3390/biomedicines9080852. 34440056 PMC8389626

[116] B. J. Monk , J. T. Kauderer , K. M. Moxley , et al., “A Phase II Evaluation of Elesclomol Sodium and Weekly Paclitaxel in the Treatment of Recurrent or Persistent Platinum‐Resistant Ovarian, Fallopian Tube or Primary Peritoneal Cancer: An NRG Oncology/Gynecologic Oncology Group Study, Gynecologic Oncology 151 (2018): 422–427, 10.1016/j.ygyno.2018.10.001. 30309721 PMC6392076

[117] S. R. Bareggi and U. Cornelli , “Clioquinol: Review of Its Mechanisms of Action and Clinical Uses in Neurodegenerative Disorders,” CNS Neuroscience & Therapeutics 18 (2012): 41–46, 10.1111/j.1755-5949.2010.00231.x. 21199452 PMC6493473

[118] D. Noh , H. Lee , S. Lee , et al., “Copper‐Based Nanomedicines for Cuproptosis‐Mediated Effective Cancer Treatment,” Biomaterials Research 28 (2024): 0094, 10.34133/bmr.0094. 39430913 PMC11486892

[119] J. Lu , Y. Miao , and Y. Li , “Cuproptosis: Advances in Stimulus‐Responsive Nanomaterials for Cancer Therapy,” Advanced Healthcare Materials 13 (2024): 2400652, 10.1002/adhm.202400652. 38622782

[120] L. Mao , J. Lu , X. Wen , et al., “Cuproptosis: Mechanisms and Nanotherapeutic Strategies in Cancer and beyond,” Chemical Society Reviews 54 (2025): 6282–6334, 10.1039/D5CS00083A. 40433941

[121] S. Lu , Y. Li , and Y. Yu , “Glutathione‐Scavenging Celastrol‐Cu Nanoparticles Induce Self‐Amplified Cuproptosis for Augmented Cancer Immunotherapy,” Advanced Materials (deerfield Beach, Fla.) 36 (2024): 2404971, 10.1002/adma.202404971. 38935977

[122] C. Xiao , X. Wang , S. Li , et al., “A Cuproptosis‐Based Nanomedicine Suppresses Triple Negative Breast Cancers by Regulating Tumor Microenvironment and Eliminating Cancer Stem Cells,” Biomaterials 313 (2025): 122763, 10.1016/j.biomaterials.2024.122763. 39180917

[123] N. Wang , Y. Liu , D. Peng , et al., “Copper‐Based Composites Nanoparticles Improve Triple‐Negative Breast Cancer Treatment with Induction of Apoptosis‐Cuproptosis and Immune Activation,” Advanced Healthcare Materials 13 (2024): 2401646, 10.1002/adhm.202401646. 39001628

[124] H. Zhang , F. Chen , W. Cheng , et al., “Dynamic Polyphenol Nanoparticles Boost Cuproptosis‐Driven Metalloimmunotherapy of Breast Cancer,” Nano Today 58 (2024): 102442, 10.1016/j.nantod.2024.102442.

[125] Y. Sun , E. Li , W. Zhong , et al., “GSH/pH‐Responsive Copper‐Based Cascade Nanocomplexes Inducing Immunogenic Cell Death through Cuproptosis/Ferroptosis/Necroptosis in Oral Squamous Cell Carcinoma,” Materials Today Bio 30 (2025): 101434, 10.1016/j.mtbio.2024.101434. PMC11750277 39839490

[126] G. Zhu , Y. Xie , J. Wang , et al., “Multifunctional Copper‐Phenolic Nanopills Achieve Comprehensive Polyamines Depletion to Provoke Enhanced Pyroptosis and Cuproptosis for Cancer Immunotherapy,” Advanced Materials (deerfield Beach, Fla.) 36 (2024): 2409066, 10.1002/adma.202409066. 39285820

[127] J.‐E. Cun , Z. He , X. Fan , et al., “Copper‐Based Bio‐Coordination Nanoparticle for Enhanced Pyroptosis‐Cuproptosis Cancer Immunotherapy through Redox Modulation and Glycolysis Inhibition,” Small (weinheim an Der Bergstrasse, Germany) 21 (2025): 2409875, 10.1002/smll.202409875. 39757406

[128] Y. Wan , J. Chen , J. Li , et al., “Cu0‐Based Nanoparticles Boost Anti‐Tumor Efficacy via Synergy of Cuproptosis and Ferroptosis Enhanced by Cuproptosis‐Induced Glutathione Synthesis Disorder,” Colloids and Surfaces B: Biointerfaces 245 (2025): 114196, 10.1016/j.colsurfb.2024.114196. 39243710

[129] J. Wang , J. Wang , J. Zhang , et al., “Bimetallic Chitosan/Hyaluronic Acid Nanoparticles Self‐Amplify Ferroptosis/Cuproptosis in Triple‐Negative Breast Cancer,” International Journal of Biological Macromolecules 308 (2025): 142535, 10.1016/j.ijbiomac.2025.142535. 40174837

[130] W. Zhang , Z. Chen , C. Xiong , et al., “A Peptide‐Copper Self‐Assembled Nanoparticle for Enhanced Cuproptosis by Metabolic Reprogramming in Tumor Cells,” ACS Nano 18 (2024): 34244–34256, 10.1021/acsnano.4c12123. 39625713

[131] Z. Wang , Q. Ye , S. Yu , and B. Akhavan , “Poly Ethylene Glycol (PEG)‐Based Hydrogels for Drug Delivery in Cancer Therapy: A Comprehensive Review,” Advanced Healthcare Materials 12 (2023): 2300105, 10.1002/adhm.202300105. 37052256 PMC11468892

[132] J. Li , N. Du , Y. Tan , et al., “Conjugated Polymer Nanoparticles Based on Copper Coordination for Real‐Time Monitoring of pH‐Responsive Drug Delivery,” ACS Applied Bio Materials 4 (2021): 2583–2590, 10.1021/acsabm.0c01564. 35014375

[133] L.‐C. Wang , L.‐C. Chang , W.‐Q. Chen , et al., “Atomically Dispersed Golds on Degradable Zero‐Valent Copper Nanocubes Augment Oxygen Driven Fenton‐Like Reaction for Effective Orthotopic Tumor Therapy,” Nature Communications 13 (2022): 7772, 10.1038/s41467-022-35515-8. PMC9755215 36522345

[134] Z. Xu , C. Wang , Y. Ying , et al., “Dual‐Pronged Metal Ion Interference Therapy: Copper–zinc Bimetallic Sulfide Nanoparticles Synergistically Disrupt Redox Homeostasis for Enhanced Tumor Treatment,” Rare Metals 44 (2025): 8744–8756, 10.1007/s12598-025-03540-3.

[135] Y. Song , G. Zhan , and S.‐F. Zhou , “Design of Near Infrared Light‐Powered Copper Phyllosilicate Nanomotors for Cuproptosis‐Based Synergistic Cancer Therapy,” Advanced Functional Materials 34 (2024): 2314568, 10.1002/adfm.202314568.

[136] J. Zhang , L. Peng , Y. Hao , et al., “Biodegradable CuMoO4 Nanodots with Multienzyme Activities for Multimodal Treatment of Tumor,” Advanced Healthcare Materials 12 (2023): 2300167, 10.1002/adhm.202300167. 37223944

[137] M. T. Khulood , U. S. Jijith , P. P. Naseef , et al., “Advances in Metal‐Organic Framework‐Based Drug Delivery Systems,” International Journal of Pharmaceutics 673 (2025): 125380, 10.1016/j.ijpharm.2025.125380. 39988215

[138] S. Hatami , K. Chahrour , J. E. Fakhouri , et al., “Metal–Organic Framework‐Based Drug Delivery Systems for Cancer Therapy: A Review,” International Journal of Molecular Sciences 27 (2026): 1548, 10.3390/ijms27031548. 41683966 PMC12897799

[139] H. D. Lawson , S. P. Walton , and C. Chan , “Metal–Organic Frameworks for Drug Delivery: A Design Perspective,” ACS Applied Materials & Interfaces 13 (2021): 7004–7020, 10.1021/acsami.1c01089. 33554591 PMC11790311

[140] K. Li , L. Wu , H. Wang , et al., “Apoptosis and Cuproptosis Co‐Activated Copper‐Based Metal‐Organic Frameworks for Cancer Therapy,” Journal of Nanobiotechnology 22 (2024): 546, 10.1186/s12951-024-02828-3. 39237931 PMC11378619

[141] J. Deng , H. Zhuang , S. Shao , et al., “Mitochondrial‐Targeted Copper Delivery for Cuproptosis‐Based Synergistic Cancer Therapy,” Advanced Healthcare Materials 13 (2024): 2304522, 10.1002/adhm.202304522. 38530073

[142] T. M. Chang , S. Sinharay , A. V. Astashkin , and E. Tomat , “Prodigiosin Analogue Designed for Metal Coordination: Stable Zinc and Copper Pyrrolyldipyrrins, Inorganic Chemistry 53 (2014): 7518–7526, 10.1021/ic5008439. 25008284 PMC4106694

[143] T. Paul , T. K. Bandyopadhyay , A. Mondal , et al., “A Comprehensive Review on Recent Trends in Production, Purification, and Applications of Prodigiosin,” Biomass Conversion and Biorefinery 12 (2022): 1409–1431, 10.1007/s13399-020-00928-2.

[144] M. S. Melvin , J. T. Tomlinson , G. Park , et al., “Influence of the A‐Ring on the Proton Affinity and Anticancer Properties of the Prodigiosins,” Chemical Research in Toxicology 15 (2002): 734–741, 10.1021/tx025507x. 12018996

[145] M. Liu , H. Miao , T. Ye , et al., “Precision Cuproptosis Activation via HER2‐Targeted Bimetallic MOF Nanoreactors Breaks Metabolic Adaptation in Aggressive Breast Cancers,” Advanced Functional Materials 36 (2026): e12905, 10.1002/adfm.202512905.

[146] J. Zhong , X. Zheng , Y. Wen , et al., “ *In Situ* Sacrificial Growth of Metastable Copper‐Enriched Nanomedicine for Cuproptosis‐Based Synergistic Cancer Therapy,” Chemical Engineering Journal (lausanne, Switzerland: 1996) 474 (2023): 145795, 10.1016/j.cej.2023.145795.

[147] J. Bing , B. Zhou , M. Chen , et al., “Nanomedicine‐Enabled Concurrent Regulations of ROS Generation and Copper Metabolism for Sonodynamic‐Amplified Tumor Therapy,” Biomaterials 318 (2025): 123137, 10.1016/j.biomaterials.2025.123137. 39884132

[148] B. Li , X. Wang , L. Chen , et al., “Ultrathin Cu‐TCPP MOF Nanosheets: A New Theragnostic Nanoplatform with Magnetic Resonance/Near‐Infrared Thermal Imaging for Synergistic Phototherapy of Cancers,” Theranostics 8 (2018): 4086–4096, 10.7150/thno.25433. 30128038 PMC6096389

[149] Q. X. Huang , J. L. Liang , Q. W. Chen , et al., “Metal‐Organic Framework Nanoagent Induces Cuproptosis for Effective Immunotherapy of Malignant Glioblastoma,” Nano Today 51 (2023): 101911, 10.1016/j.nantod.2023.101911.

[150] J. Zhang , M. Han , J. Zhang , et al., “Syphilis Mimetic Nanoparticles for Cuproptosis‐Based Synergistic Cancer Therapy via Reprogramming Copper Metabolism,” International Journal of Pharmaceutics 640 (2023): 123025, 10.1016/j.ijpharm.2023.123025. 37164186

[151] H. Tian , J. Duan , B. Li , et al., “Clinical Chemotherapeutic Agent Coordinated Copper‐Based Nanoadjuvants for Efficiently Sensitizing Cancer Chemo‐Immunotherapy by Cuproptosis‐Mediated Mitochondrial Metabolic Reprogramming,” Advanced Functional Materials 33 (2023): 2306584, 10.1002/adfm.202306584.

[152] M. Xu , H. Chen , G. Zhu , et al., “Spiky Metal‐Organic Framework Nanosystem for Enhanced Cuproptosis‐Mediated Cancer Immunotherapy,” Nano Today 56 (2024): 102231, 10.1016/j.nantod.2024.102231.

[153] Z. Wang , M. Han , Y. Wang , et al., “UiO‐66 MOFs‐Based “Epi‐Nano‐Sonosensitizer” for Ultrasound‐Driven Cascade Immunotherapy against B‐Cell Lymphoma,” ACS Nano 19 (2025): 6282–6298, 10.1021/acsnano.4c15761. 39920081

[154] Z. Wu , M. Gao , Q. Li , et al., “Copper Metal‐Organic Framework‐Based Multifaceted Strategy for Boosting Cancer Therapy via Synergistic Cuproptosis and Disulfidptosis,” Biomaterials 325 (2026): 123592, 10.1016/j.biomaterials.2025.123592. 40780141

[155] Y.‐E. Wang , H. Yang , S. Zhao , et al., “Dextran‐Engineered Cu‐MOF Nanozyme with Multi‐Enzyme Mimetic Cascade for Cuproptosis‐Enhanced Synergistic Therapy in Triple‐Negative Breast Cancer,” International Journal of Biological Macromolecules 322 (2025): 146920, 10.1016/j.ijbiomac.2025.146920. 40819751

[156] G. Wan , J. Chen , Y. Zhou , et al., “Light‐Cured Millineedle Platform Delivers “Nano‐Pomegranate” for Combinatorial Electrodynamic Therapy and Cuproptosis against Oral Carcinoma,” Bioactive Materials 53 (2025): 718–736, 10.1016/j.bioactmat.2025.07.032. 40801019 PMC12341573

[157] Y. Xia , M. Gu , J. Wang , et al., “Tumor Microenvironment‐Activated, Immunomodulatory Nanosheets Loaded with Copper(II) and 5‐FU for Synergistic Chemodynamic Therapy and Chemotherapy,” Journal of Colloid and Interface Science 653 (2024): 137–147, 10.1016/j.jcis.2023.09.042. 37713912

[158] Y. Zhong , Y. Zhang , W. Peng , et al., “Cuproptosis‐Based Nanoparticles for Cascade Reaction to Boost Radioimmunotherapy,” Colloids and Surfaces B: Biointerfaces 255 (2025): 114888, 10.1016/j.colsurfb.2025.114888. 40543186

[159] M. Li , Z. Liu , D. Peng , et al., “Multifunctional Porous Organic Polymer‐Based Hybrid Nanoparticles for Sonodynamically Enhanced Cuproptosis and Synergistic Tumor Therapy,” Acta Biomaterialia 196 (2025): 350–363, 10.1016/j.actbio.2025.02.045. 39993518

[160] Y. Li , J. Liu , Y. Chen , et al., “Nanoparticles Synergize Ferroptosis and Cuproptosis to Potentiate Cancer Immunotherapy,” Advanced Science 11 (2024): 2310309, 10.1002/advs.202310309. 38477411 PMC11187894

[161] J. Wang , L.‐Z. Luo , D.‐M. Liang , et al., “Progress in the Research of Cuproptosis and Possible Targets for Cancer Therapy,” World Journal of Clinical Oncology 14 (2023): 324–334, 10.5306/wjco.v14.i9.324. 37771632 PMC10523190

[162] C. Zhang , T. Huang , and L. Li , “Targeting Cuproptosis for Cancer Therapy: Mechanistic Insights and Clinical Perspectives,” Journal of Hematology & Oncology 17 (2024): 68, 10.1186/s13045-024-01589-8. 39152464 PMC11328505

[163] L. Xu , X. Cao , Y. Deng , et al., “Cuproptosis‐Related Genes and Agents: Implications in Tumor Drug Resistance and Future Perspectives,” Frontiers in Pharmacology 16 (2025), 10.3389/fphar.2025.1559236. PMC12095339 40406488

